# Concurrent excitation and detection in magnetic resonance using voltage-controlled oscillators

**DOI:** 10.1126/sciadv.aeg6581

**Published:** 2026-09-16

**Authors:** Zhibin Zhao, Michal Kern, Weiyi Zhang, Qing Yang, Katja Drerup, Felix Schuderer, Samuel Wazynski, Bernhard Blümich, Klaus Lips, Klaus Scheffler, Jens Anders

**Affiliations:** ^1^ Institute of Smart Sensors, University of Stuttgart, 70569 Stuttgart, Germany.; ^2^ Institute of Microstructure Technology, Karlsruhe Institute of Technology, 76344 Eggenstein-Leopoldshafen, Germany.; ^3^ Institut für Technische und Makromolekulare Chemie, RWTH Aachen University, 52074 Aachen, Germany.; ^4^ Berlin Joint EPR Laboratory and EPR4Energy, Department Spins in Energy Conversion and Quantum Information Science (ASPIN), Helmholtz-Zentrum Berlin für Materialien und Energie GmbH, Berlin, Germany.; ^5^ Berlin Joint EPR Laboratory, Fachbereich Physik, Freie Universität Berlin, Berlin, Germany.; ^6^ High-Field Magnetic Resonance Center, Max Planck Institute for Biological Cybernetics, 72076 Tübingen, Germany.; ^7^ Department of Biomedical Magnetic Resonance, Eberhard-Karls University, 72076 Tübingen, Germany.; ^8^ Institute for Microelectronics Stuttgart (IMS Chips), 70569 Stuttgart, Germany.

## Abstract

Dead time following radio frequency excitation limits pulsed magnetic resonance (MR) experiments by obscuring early-time spin dynamics and short-lived signals. We demonstrate continuous concurrent excitation and detection using two oscillator-based MR detectors: a high-frequency (477 megahertz) injection-locked voltage-controlled oscillator (VCO) array enabling a scalable segmented-coil architecture and a low-frequency (17 megahertz) single-coil VCO. Both detectors are embedded in custom-designed phase-locked loops, enabling phase-coherent multipulse operation. Using a planar segmented coil driven by a chip-integrated VCO array operating at 477-megahertz proton frequency, we perform conventional Fourier transform nuclear MR, continuously monitor driven Rabi oscillations throughout the excitation pulse, and observe the immediate onset of free-induction decay, all without dedicated transmit-receive switches or receiver gating. We further demonstrate phase-coherent, phase-cycled multipulse operation, including a Hahn echo sequence realized on a second system operating at 17 megahertz. Together, these results demonstrate the general applicability and versatility of the oscillator-based detection principle across pulse sequences and operating frequencies.

## INTRODUCTION

**Fig. 1.**
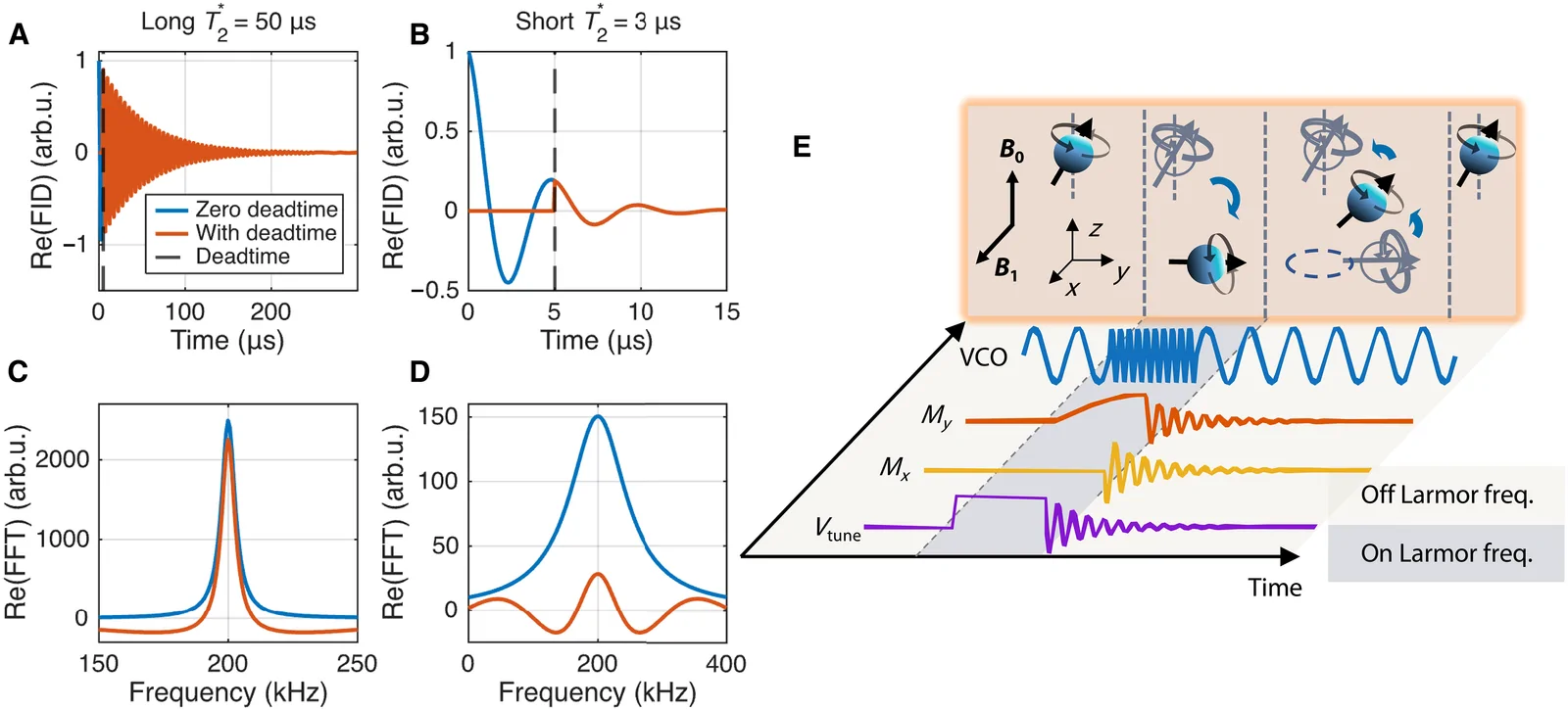
Conceptual illustration of dead time–free detection using oscillator-based MR. (**A**) Simulated FID acquired with zero dead time (blue) and with a finite receiver dead time of 5 μs (orange) with a comparatively long transverse relaxation time *T*_2_^*^. (**B**) Simulated FID with a *T*_2_^*^ short compared to the dead time, highlighting the loss of early signal during the dead time interval. (**C**) Fourier spectra of the FID in (A), with only a modest loss of signal intensity but a notable phase shift caused by the dead time. (**D**) Fourier spectrum of the FID in (B) illustrating the notable linewidth distortion and signal loss caused by dead time, which becomes severe for short-lived signals. (**E**) Principle of oscillator-based detection: a voltage-controlled oscillator (VCO) drives a coil continuously, while the VCO frequency is switched on and off the Larmor frequency for pulsed excitation. The spin-induced back-action modulates the oscillator frequency and amplitude. In the PLL-referenced implementation used in this paper, the frequency modulation (dispersive transverse magnetization component, *M_x_*) is converted into a baseband tuning voltage signal *V*_tune_, enabling continuous observation of spin dynamics during excitation and free precession. The amplitude modulation (absorptive magnetization component, *M_y_*) is indicated for completeness but not used for detection in this work. arb.u., arbitrary units.

Nuclear magnetic resonance (NMR) and related magnetic resonance (MR) techniques such as electron paramagnetic resonance (EPR) are foundational tools in physics, chemistry, materials science, and medicine (*1*). All MR modalities rely on detecting weak, time-dependent signals generated by precessing spin ensembles in response to a high-frequency excitation, typically in the radio frequency (RF) to microwave frequency range. In conventional pulsed MR systems, however, acquisition cannot begin immediately after a pulse. A finite delay—commonly termed dead time*—*arises because the same resonant structure must accommodate both high-power transmission and very-low-noise reception. The transmit/receive (T/R) switching networks, preamplifier recovery, and resonator ring-down together impose dead times ranging from tens of nanoseconds to several microseconds, during which a substantial and often irrecoverable fraction of the free-induction decay (FID) is lost (*2*–*4*). Because the earliest part of the FID contains the highest signal amplitude and fastest dynamics, this truncation not only reduces sensitivity but also leads to distortion of the reconstructed spectrum, including line broadening, baseline errors, and loss of short-lived components (Fig. 1, A to C). For samples with short transverse relaxation times (*T*_2_^*^), such as solids, paramagnetic samples, or environments with strong susceptibility or field gradients, this loss can preclude detection altogether (*5*–*8*). Dead time also limits time resolution in studies of ultrafast spin dynamics and constrains pulse-sequence design in NMR, EPR, and MR imaging (*9*).

Multiple hardware strategies aim to reduce dead time. Fast PIN-diode or GaAs T/R switches (*9*, *10*), active *Q*-dampers (*11*), and limiters (*4*, *12*) shorten the transition from excitation to detection, yet residual nonlinearities and coil ring-down persist. Architectures that attempt concurrent excitation and acquisition instead actively cancel the transmitter leakage signal in the receive path. However, the difference between the transmit and receive signals can reach up to 100 dB (*13*), and because the achievable isolation depends on coil loading, practical implementations require continuous, real-time feedback control of the cancelation circuitry, adding hardware complexity. Related approaches such as continuous SWIFT (*14*) instead acquire signal throughout a swept-frequency excitation and separate the resulting transmitter leakage from the spin signal by model-based postprocessing. These methods require only modest (∼40 dB) transmitter-receiver isolation and minimal hardware modification. However, as with active cancelation approaches, the transmitter leakage and the spin signal are processed by the same receiver chain. Consequently, the achievable bandwidth and signal-to-noise ratio (SNR) remain fundamentally coupled to the available transmitter-receiver isolation and the receiver’s dynamic range. Miniaturized coils reduce stored magnetic energy and, hence, the ring-down time but at the expense of reduced concentration sensitivity (*15*). Pulsed EPR faces constraints analogous to, or even more stringent than, those of NMR, due to higher operating frequencies and high-*Q* resonators, with near–zero–dead time performance achieved only in specialized, high-power, and instrumentally intensive setups (*16*).

An alternative approach is oscillator-based MR detection (*17*–*21*). Instead of treating excitation and detection as two separate modes gated in time, a self-oscillating circuit can continuously drive and sense the sample. The interaction between an LC oscillator and a precessing spin ensemble arises from the bidirectional coupling between the LC tank inductor and the sample magnetization. The oscillating current in the inductor generates the transverse *B*_1_ field that excites the spins, while the time-dependent magnetization induces an electromotive force (emf) in the same conductor. This back-action perturbs the oscillator’s instantaneous frequency and amplitude, yielding a direct electrical readout of the spin dynamics at the oscillator’s output. A detailed discussion of the origin of both signal and noise in oscillator-based MR detection can be found in section S3. In oscillator-based pulsed MR, excitation is achieved by briefly shifting the oscillator frequency to the Larmor frequency to perform the required spin manipulation and then returning the oscillator to an off-resonance condition while recording the signal (see Fig. 1D). Crucially, the oscillator remains running throughout this process, and the LC tank stores approximately the same amount of energy when operating at different frequencies, causing the oscillation amplitude to change only weakly. As a result, virtually no resonator ring-down occurs after the frequency hops. Because the oscillator remains active at all times, no T/R switching is required, and no high-power amplifier or low-noise receive chain must recover from overload. Oscillator-based detectors are thus intrinsically suited for low–dead time or even dead time–free operation, and their compatibility with on-chip integration opens the door to scalable sensor arrays (*20*, *22*).

Prior oscillator-based MR demonstrations have highlighted both the promise and the challenges of this detection paradigm. Early implementations in NMR and EPR used free-running oscillators in continuous-wave configurations, enabling simultaneous excitation and readout and thereby avoiding pulsed T/R switching dead time (*17*–*19*). In principle, free-running oscillators can maintain excellent short-term phase coherence; however, their absolute phase and frequency drift with environmental conditions, particularly temperature. For routine pulsed NMR and EPR experiments, where coherent averaging, phase cycling, and multipulse timing require a stable global phase reference, this drift complicates practical operation unless stabilization is used. Subsequent work introduced oscillators stabilized using phase-locked loops (PLLs) that provide a well-defined reference phase and frequency (*23*–*25*). However, when used in pulsed measurements, the finite loop bandwidth and nonlinear settling dynamics of the PLL can introduce transient disturbances following excitation pulses, effectively reintroducing dead time.

Two recent developments attempted to bridge this gap. First, a pulsed EPR implementation using an on-chip high-bandwidth PLL enabled continuous detection of Rabi oscillations during the excitation pulse and FIDs thereafter (*26*). Although the PLL was substantially faster than its predecessors, the experiment exhibited clearly visible PLL settling transients following strong excitation pulses. These transients are especially detrimental at EPR timescales, where spin dynamics evolve over tens of nanoseconds. Second, a first proof-of-concept voltage-controlled oscillator (VCO)–NMR demonstration used a single VCO embedded in a relatively slow PLL and reported that the method can in principle be dead time free (*20*). In practice, however, PLL settling transients of several microseconds were apparent in the measurements, and the work explicitly acknowledged that eliminating these transients would require substantially faster loop dynamics (*20*).

In the work reported here, we overcome both limitations. We introduce an injection-locked oscillator array referenced by a PLL whose loop dynamics are engineered to be much faster than the relevant NMR spin evolution timescales. As a result, the PLL remains phase stable without introducing appreciable settling artifacts for conventional NMR experiments. This enables practical dead time–free detection for a broad class of NMR experiments without requiring any cancelation, switching, or postprocessing beyond standard baseline and phase correction. Using a second detector operating at a substantially lower frequency, we use phase-cycled pulse sequences to demonstrate the general applicability and versatility of this approach.

In addition, we show that the residual PLL transient response following an excitation pulse is completely deterministic. In a controlled electrical experiment, we demonstrate that the transient can be recorded at an off-resonance frequency and subtracted from subsequent measurements, enabling complete recovery of the signal during the excitation pulse and the complete FID signal with zero measurable dead time and no measurable artifacts. In a phase-cycled Hahn echo experiment on a real spin signal, the same procedure yields a substantial reduction; however, some of the transient artifacts remain. These artifacts, however, arise from identifiable, addressable sources (see Results) rather than from any fundamental limitation of the approach. Together with the presented theoretical considerations, these results establish that at the fundamental level, oscillator-based MR detectors can combine continuous operation, phase coherence, and dead time–free operation at the cost of a relatively small, quantifiable electronic noise penalty intrinsic to the detection principle.

Using our custom-designed VCO systems, we demonstrate (i) Fourier transform NMR (FT-NMR) of a multiline spectrum, (ii) continuous monitoring of Rabi oscillations and immediate FID onset after the pulse, (iii) phase-coherent multipulse operation, and (iv) complete and artifact-free early-time signal recovery via deterministic PLL transient subtraction in a purely electrical experiment.

## RESULTS

### High-field VCO array system design and principle

**Fig. 2.**
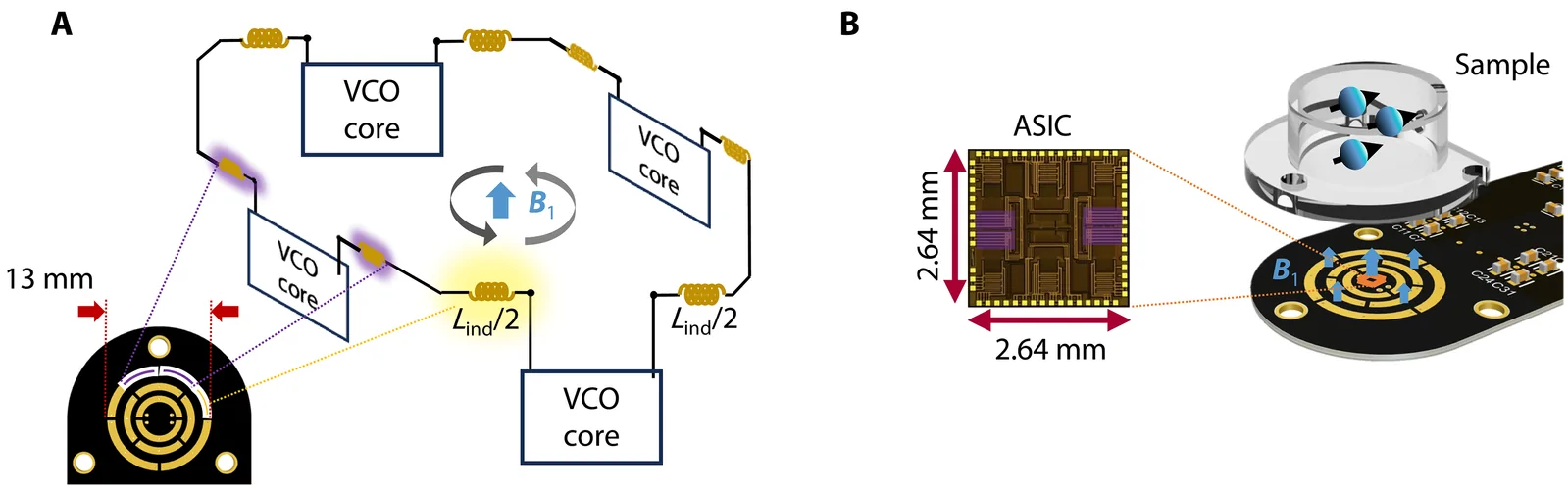
Overview of the implemented oscillator-based pulsed MR system. (**A**) Simplified schematic of a planar segmented coil on a PCB driven by multiple injection-locked VCO cores. Each coil segment with an inductance *L*_ind_/2 forms a part of the LC tank of a VCO, generating the transverse excitation field *B*_1_. (**B**) Physical implementation of the NMR probe head, including the fabricated application-specific integrated circuit (ASIC) containing the VCO cores and a three-dimensional (3D) printed liquid sample holder positioned above the coil.

The custom-designed MR detector presented here achieves phase-coherent, dead time–free MR detection within a compact, scalable architecture. The system combines a custom-designed printed circuit board (PCB)–based planar segmented coil array, a custom-designed integrated VCO array implemented in a 0.35-μm high-voltage complementary metal-oxide semiconductor (HV CMOS) technology, and an off-chip PLL that defines the VCO frequency and phase from a stable reference signal. Figure 2 provides a simplified overview of the hardware used in our measurements.

The planar coil array consists of three concentric segmented rings. Each ring is electrically subdivided into smaller segments, each driven and sensed by a corresponding set of VCO cores. Segmentation enables scaling the overall sensitive area while maintaining an in-phase current distribution across the structure; the VCO cores enforce the desired phase relationship in each segment so that the magnetic field pattern closely resembles the low-frequency magnetic field distribution of the corresponding unsegmented coil. This is achieved because the segmentation enforces a current and electric field distribution in the segmented coil that prevents radiative losses. The segmented coil concept for MR applications was first introduced by Chu *et al.* (*27*) in an injection-locked VCO array operating at 263 GHz for continuous-wave EPR detection, where the goal was to increase sensitive volume at very high carrier frequency and pulsed operation was not considered. In contrast, the presented implementation operates around 477 MHz (corresponding to a static magnetic field *B*_0_ = 11.2 T for protons) and is optimized for pulsed MR detection with effectively zero dead time. The overall coil size of 13 mm (sensitive volume of ∼575 μl) improves concentration sensitivity for relaxometry and spectroscopy and expands the usable field of view for imaging. The chosen operating frequency is not intrinsic to the detection concept but is determined by the LC tank parameters and can be readily adjusted for other target operating frequencies, as demonstrated in the low-field implementation in the “Phase-cycled Hahn echo” section.

Each coil segment is connected to two VCO cores fabricated in the same 0.35-μm HV CMOS process on a single chip. The cores provide sufficient voltage headroom for relatively large-swing oscillation up to 25 V, allowing for sufficient *B*_1_ field strengths and a tuning range of ∼10 MHz. The VCO tuning voltage (*V*_tune_) sets the instantaneous oscillation frequency of all VCO cores. All of the VCO cores are injection locked to ensure phase alignment, and the entire array is referenced to the external PLL. This locking scheme suppresses beat artifacts and yields a single, phase-stable oscillation frequency for the entire coil array. Details of the VCO cores and PLL structure can be found in section S1.

To preserve phase coherence between repeated excitation cycles, we connect the VCO array output to an off-chip PLL comprising a phase-frequency detector, a loop filter, and a stable, low-noise reference signal generator. The PLL enforces frequency and phase coherence with respect to this reference, which is required for coherent averaging and other phase-sensitive pulsed experiments. Following large frequency jumps used for excitation or detection, the PLL exhibits a transient settling behavior as the loop reestablishes lock. For a given frequency step and loop configuration, this transient is highly reproducible, enabling the deterministic subtraction of the purely electrical response and thus the removal of artifacts in the reconstructed MR signal, effectively enabling zero–dead time detection.

During measurement, the VCO array excites the sample via an RF magnetic field produced by the oscillation current in the tank inductor, and the spin-related sample magnetization induces an emf in the tank inductor that modulates both the VCO’s oscillation frequency and amplitude. Within its loop bandwidth, the PLL feedback action causes the VCO frequency to track the PLL reference, causing the tuning voltage *V*_tune_ to track the frequency modulation with opposite polarity, effectively canceling the spin signal in the VCO output. That is, the PLL converts the spin-induced frequency modulation, proportional to the dispersive component of the spin signal, into a baseband voltage proportional to the temporal evolution of the spins while keeping the carrier phase-locked to the reference.

### Fourier transform NMR

**Fig. 3.**
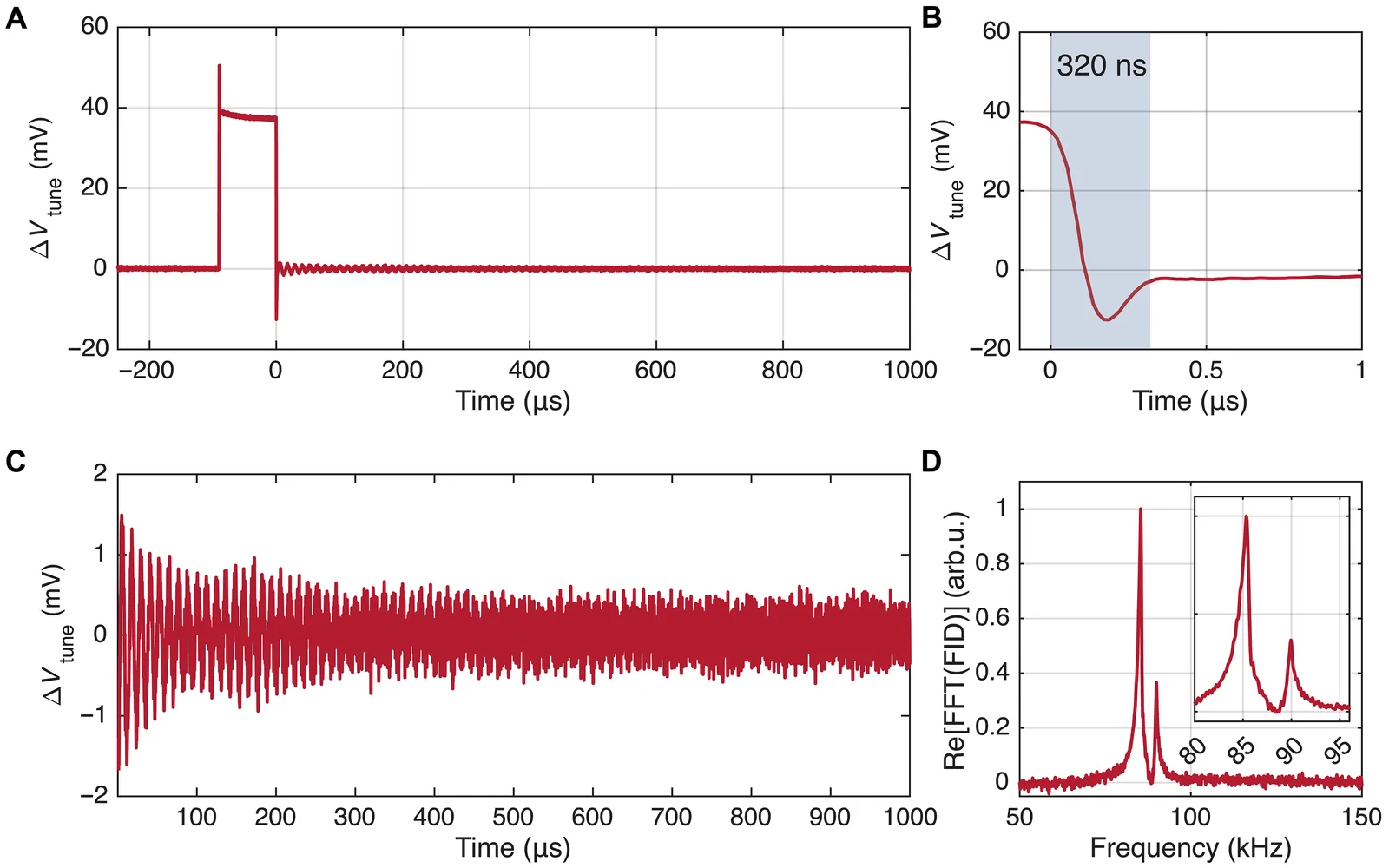
Pulsed NMR measurements on acetic acid. (**A**) Raw time domain data of *V*_tune_ during a single-pulsed NMR experiment. (**B**) Detailed view of the PLL settling behavior (blue section) measured to be 320 ns. (**C**) Detailed view of the FID. (**D**) Fast Fourier transform (FFT) spectrum of the FID, confirming the typical shape for acetic acid with the peak separation of ∼4.6 kHz (9.6 ppm). The inset is showing a detail of the spectral lines.

We first evaluated the performance of the presented injection-locked VCO detector in a standard pulsed FT-NMR experiment using a liquid acetic acid sample, whose FID exhibits a moderately long transverse relaxation time *T*_2_^*^ (limited by magnetic field inhomogeneity) and a well-defined spectral pattern. Figure 3A shows the raw time-domain output of *V*_tune_ acquired during a single 90° pulse-acquire sequence (see section S2 for details of calibration). Immediately after the excitation pulse, the PLL undergoes a brief settling transient, marked in blue, corresponding to the frequency step used to toggle the VCO on- and off-resonance. For the operating conditions used here, the duration of this transient is ∼320 ns (Fig. 3B), which is short compared with the *T*_2_^*^ of the sample and produces no observable distortion in the subsequent FID.

Following the transient PLL settling, the FID is recorded continuously with no detectable gap or amplitude discontinuity (Fig. 3C). No signal conditioning beyond linear baseline subtraction was applied. For spectral analysis, a conventional Fourier transform was applied to the FID portion of the time trace, starting immediately after the PLL transient. The NMR spectrum (Fig. 3D), after a zero-order phase correction to recover the usual absorption line shape, shows the expected two-line spectrum of acetic acid with a peak separation of ∼4.6 kHz [∼9.6 parts per million (ppm)], in close agreement with the reference value of ∼9.4 ppm (*28*). The linewidth is dominated by magnetic field inhomogeneity of the used magnet and not by detection artifacts, consistent with the fact that the effective dead time of the detector is substantially shorter than the intrinsic decay time of the signal. Because the transient duration is only 320 ns, starting the Fourier transform immediately after this interval corresponds to a small time-origin shift. Across a spectral width of 5 kHz (comparable to the 4.6-kHz splitting of acetic acid), this introduces a phase variation of only ∼0.6°, and even across 10 kHz, the corresponding phase variation is merely ∼1.2°. These small linear phase offsets do not produce observable line shape distortion and can be readily compensated by standard phase correction.

with the ∼1.2-mV value calculated from the VCO’s electrical characteristics and the sample’s proton concentration (see section S3.2). We note that the apparent noise level in the raw time-domain trace (Fig. 3C) is inflated by the wide oscilloscope bandwidth required to resolve the 320-ns PLL transient, which captures broadband noise from well outside the 100-kHz signal band in the displayed trace, greatly reducing the apparent time-domain SNR. When restricted to the 100-kHz NMR bandwidth, the measurement yields an SNR of 132, with a noise floor about one order of magnitude above the thermally limited value expected for the coil and oscillator circuit (see sections S3.3 and S3.4 for a detailed derivation.). The resulting spin limit of detection is $N_{\text{min}}\approx 3.4\times 10^{17}\mathrm{spins}/\sqrt{\mathrm{Hz}}$.

These measurements establish that the VCO-based detector performs conventional FT-NMR with high spectral fidelity and without dead time–induced distortions. They also confirm that, for typical NMR samples with moderately long *T*_2_^*^, the short PLL transient has no practical consequences and that the detector is effectively dead time–free in routine use.

### Concurrent excitation and real-time detection of Rabi oscillations and FIDs

To demonstrate the detector’s ability to acquire data continuously during and after an excitation pulse, we performed pulsed experiments in which the oscillator frequency ω_osc_ was offset from the Larmor frequency ω_L_ by Ω*/*2π *=* 5, 15, and 26 kHz during the excitation. Each excitation pulse was 450 μs long. From an independently measured Rabi frequency ω_R_*/*2π = 2.5 kHz (see section S2), we infer a nominal *B*_1_ field amplitude of ∼59 μT. For off-resonant excitation with detuning Ω, the spins undergo coherent rotation at an effective Rabi frequency $\omega_{\text{eff}}=\sqrt{\omega_{R}^{2}+\Omega^{2}}$. For the detunings used here, this results in expected Rabi frequencies of ∼5.6, 15.2, and 26.1 kHz, respectively. The associated transverse magnetization induces an emf in the tank, which modulates the VCO frequency. This modulation is tracked by the PLL and appears as a baseband signal in the control voltage *V*_tune_. We note that the dispersive component of the spin signal measured here vanishes during the pulse for on-resonance excitation (Ω = 0) (*26*).

**Fig. 4.**
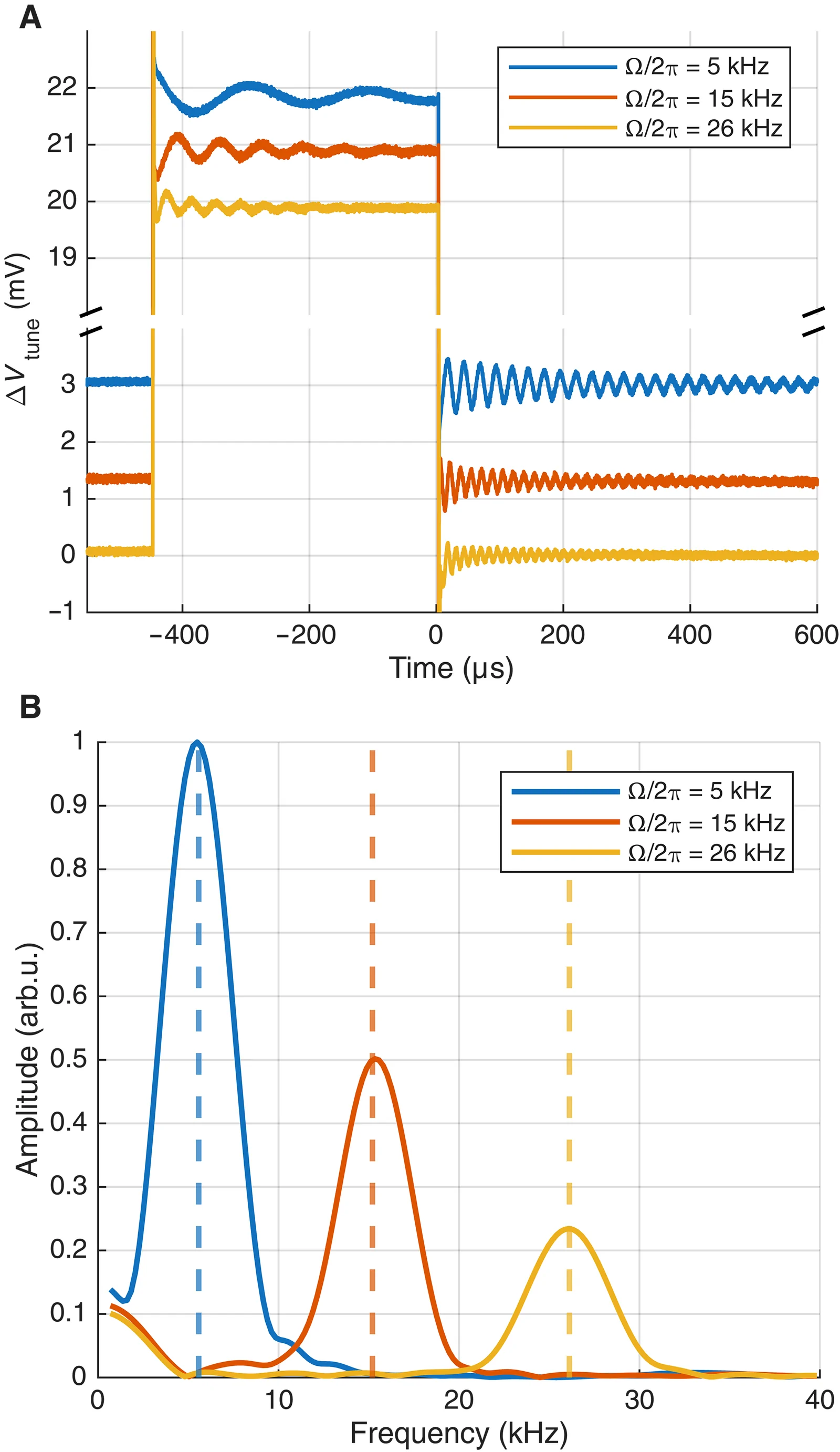
Continuous detection of driven Rabi oscillations and FID. (**A**) Time-domain *V*_tune_ data recorded continuously during and after excitation for three frequency offsets from the Larmor frequency. Traces are offset for clarity. (**B**) Fourier transforms of the driven portions of the traces in (A), showing peaks at the effective Rabi frequencies that increase with nominal detuning. Dashed lines mark the expected effective Rabi frequencies.

Figure 4A shows representative time-domain traces for the three detunings. Clear oscillations are observed during the drive for all offsets, and their frequency increases systematically with Ω as expected from $\omega_{\text{eff}}$. Because of the continuous acquisition, the transition from the driven regime to the subsequent FID is recorded without interruption. The observed decay of the driven Rabi oscillations is faster than that of the subsequent FID (τ_Rabi_ ≈ 185 μs versus τ_FID_ ≈ 245 μs for the 5-kHz detuned trace), consistent with an additional dephasing mechanism specific to the driven period. Alongside the decay caused by *B*_0_ inhomogeneity, spatial variation in *B*_1_ amplitude causes spin packets to evolve at different Rabi frequencies during the pulse, producing additional dephasing. This additional *B*_1_-induced dephasing vanishes after the pulse, leaving only the slower *B*_0_-limited FID decay, consistent with the measured coefficient of variation of the Rabi frequency (∼15%; see section S2).

At Δω*/*2π = 26 kHz, a slow low-frequency component is visible at the onset of the FID. This arises from the residual PLL settling following the frequency hop. The effect is relatively small, does not obscure the early-time evolution, and is both short and highly reproducible. This residual is fully removed in the zero–dead time reconstruction shown later by subtracting a prerecorded electrical baseline, demonstrating that it does not represent a fundamental limitation of the detection scheme. Fourier transforms of the driven portions (Fig. 4B) exhibit spectral peaks at the expected effective Rabi frequencies for each detuning, confirming that the detector accurately follows the coherent spin dynamics throughout the pulse.

### Phase-coherent multipulse operation

#### Steady-state free precession

To assess phase stability and coherence over repeated excitation cycles, we performed multipulse experiments in which identical excitation pulses were applied periodically while continuously recording the oscillator response. The pulse repetition time was chosen, such that transverse magnetization from previous excitations was not fully relaxed before the subsequent pulse, placing the system in a steady-state free-precession regime.

**Fig. 5.**
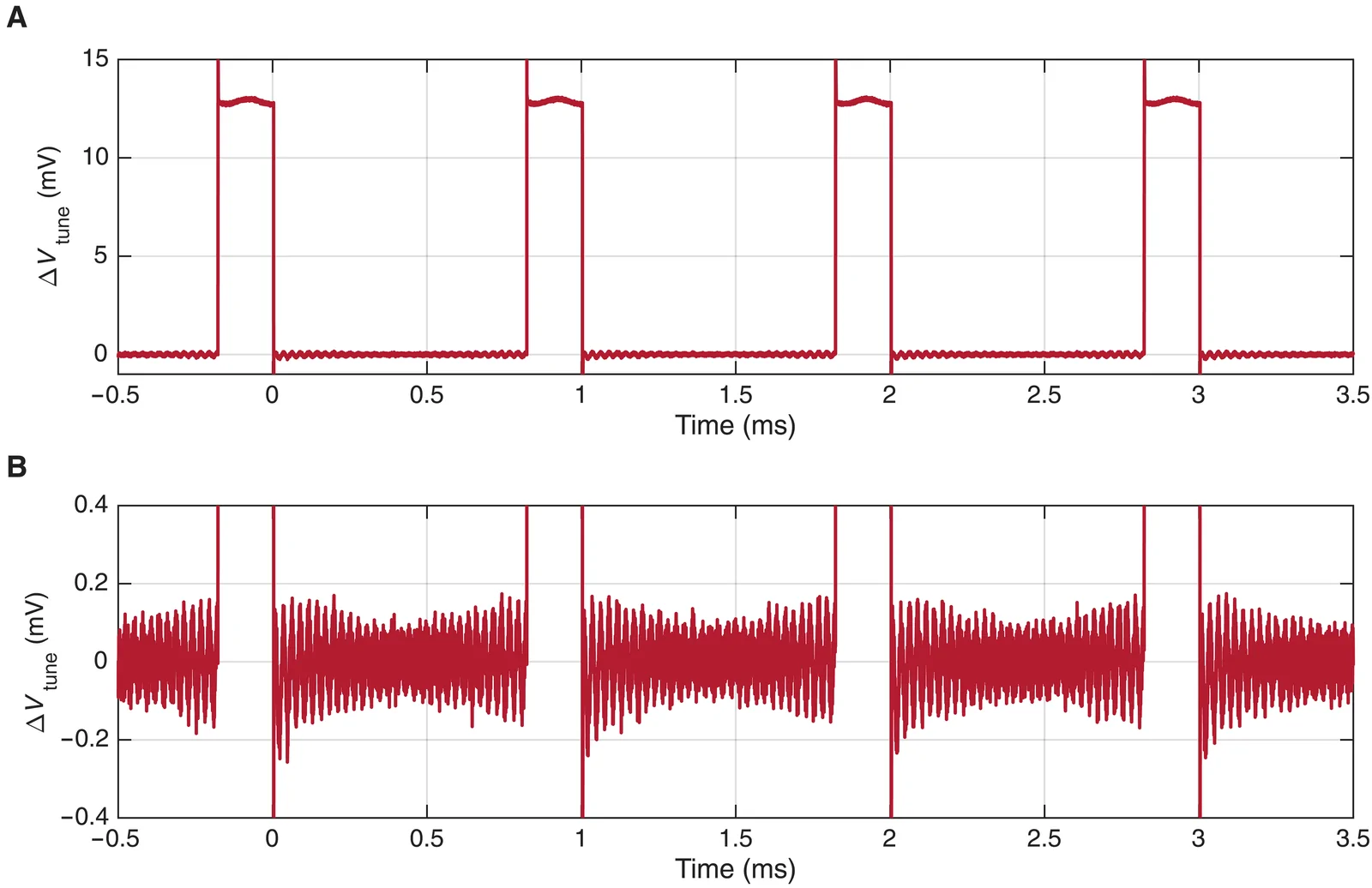
Steady-state free precession measurements. (**A**) Control voltage *V*_tune_ recorded continuously during a periodic pulse sequence, showing repeated excitation cycles without interruption of acquisition. The frequency hops used for excitation appear as step-like features, while the detector remains active throughout the sequence. (**B**) Magnified view of the interpulse intervals, revealing a reproducible oscillatory response with a stable phase relationship across successive cycles.

Figure 5 shows the resulting time-domain signal. Following an initial transient, the response converges to a reproducible steady-state waveform that persists over many pulse periods. The driven response during each pulse and the subsequent FID exhibit a stable phase relationship from cycle to cycle, demonstrating that the injection-locked VCO array maintains phase coherence across repeated excitations.

#### Phase-cycled Hahn echo

To demonstrate the generality of the oscillator-based detection principle beyond the single high-field configuration presented above and to test its compatibility with more demanding, phase-sensitive multipulse sequences, we performed phase-cycled Hahn echo measurements at 17 MHz using a second VCO-based detector. This system reuses the single VCO core architecture previously reported in (*20*), here modified with a larger-inductance coil to target this substantially lower operating frequency and referenced to a discrete, board-level PLL built from the same blocks as the high-field system. Beyond demonstrating the frequency versatility of the oscillator-based approach, the two-pulse Hahn echo sequence probes capabilities not accessed by the single-pulse experiments above. It requires precise phase control between successive excitation pulses and the detection window. Moreover, because coherent signal build-up over phase-cycled averages is only possible if this phase relationship is maintained across scans, it provides a substantially more stringent test of phase stability than previously shown measurements. We further used this measurement to test PLL-transient cancelation on a real NMR signal by subtracting a baseline transient acquired at a slightly offset, nonresonant *B*_0_ field from the acquisition containing the NMR signal.

**Fig. 6.**
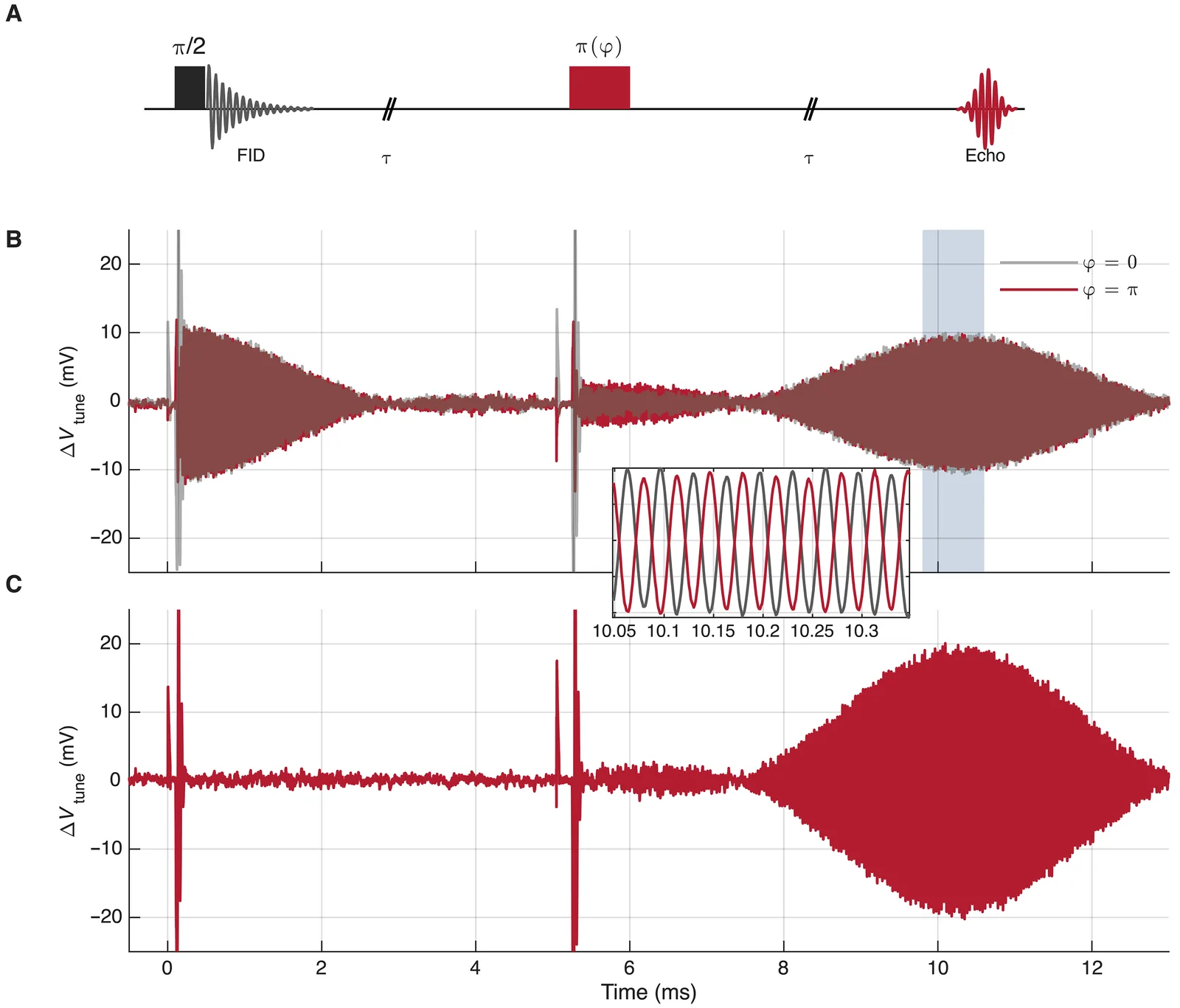
Phase-cycled Hahn echo demonstration. (**A**) Pulse sequence consisting of a π/2 excitation pulse, followed by a delay τ and a π refocusing pulse phase shifted with respect to the excitation pulse by an angle φ and a second delay τ, after which the spin echo forms. (**B**) Control voltage *V*_tune_ traces of a φ = 0 and φ = π phase shifts of the second pulse. A trace recorded at a nonresonant *B*_0_ was subtracted from both measurements. The inset shows the blue-highlighted interval in detail, highlighting the phase shift of π between the two spin echoes. The excitation (100 μs) and refocusing (200 μs) pulses were applied at times *t* = 0 ms and *t* = 5.1 ms, respectively. (**C**) *V*_tune_ trace after subtracting the φ = 0 and φ = π traces from each other. The axes in (B) and (C) are truncated to emphasize the spin signal; the amplitudes of the spike-like artifacts at the beginning and end of the pulses are specified in the text.

Figure 6A shows the applied pulse sequence: a π/2 excitation pulse, followed by a delay τ and a π refocusing pulse with a phase (φ) of either 0 or π and a second delay τ, after which the spin echo forms. As in the single-pulse experiments above, excitation and detection periods are defined by frequency hops of the VCO between the Larmor frequency and an off-resonant detection frequency. To demonstrate the compatibility of our approach with pulse sequences requiring precise phase control, we applied a two-step phase-cycling scheme. Figure 6B shows the individual φ = 0 and φ = π traces, each obtained after subtraction of a baseline acquired at a slightly offset, nonresonant *B*_0_ and averaged 32 times. The inset shows the details of the resulting spin echoes, highlighting the expected phase shift of π between the traces. Figure 6C shows the phase-cycled result obtained by subtracting the φ = 0 and φ = π traces, demonstrating the expected suppression of unwanted signals and a twofold boost of the spin-echo amplitude.

The traces in Fig. 6B show residual pulse artifacts due to imperfect cancelation visible at the start and end of both the excitation and refocusing pulses. Before this subtraction, the transients reach peak-to-peak amplitudes of ∼2 V; the residual, incompletely canceled transients have a maximum amplitude of around 67 mV and have a duration 150 μs after the end of the pulses. We attribute this imperfect cancelation to a higher bias point sensitivity of the low-frequency PLL, together with residual thermal drift of mainly off-chip components on the PCB, the off-chip LC tank, and the sample over the course of the acquisition, rather than to a fundamental limitation of the detection or cancelation principle.

To separate the purely electrical behavior from MR, we performed measurements using a synthetic NMR signal outside of a magnetic field (see section S4), where we demonstrate that under ideal conditions, the electrical response of the PLL can be perfectly subtracted, yielding a completely dead time–free FID.

## DISCUSSION

The results presented here establish oscillator-based MR detection as a viable architecture for phase-coherent pulsed NMR. By combining continuous oscillator operation with a fast PLL reference, we demonstrate uninterrupted experimental access to spin dynamics across excitation and detection, including standard FT-NMR spectroscopy, real-time observation of driven Rabi oscillations, immediate acquisition of the FID, and phase-coherent multipulse sequences. Together, these capabilities extend oscillator-based detectors beyond continuous-wave applications and into the pulsed regime.

The approach presented here differs fundamentally from concurrent excitation-and-acquisition schemes that suppress the excitation signal in the receive chain or reduce the excitation power so as not to saturate the detector. Rather than canceling a large leakage waveform at the carrier frequency, the oscillator-based detector encodes the spin response as a baseband modulation of its own oscillation frequency and amplitude. Consequently, it requires no high-bandwidth cancelation hardware. In addition, the strength of the applied *B*_1_ field is fundamentally decoupled from the intensity of the measured pulse transient, as the field is generated continuously and only its frequency is shifted. While this architecture does not eliminate all instrumental constraints of conventional pulsed MR, it shifts their origin and changes the complexity of their mitigation.

To make this shift precise, it is helpful to distinguish two distinct physical quantities governing the excitation-to-detection transition. Intrinsic dead time is the interval during which the measurement apparatus is insensitive to the signal of interest. Deterministic transient interval is the interval during which the detector is perturbed by the excitation yet still encodes the spin response in a reproducible, instrument-specific manner.

The intrinsic dead time of the oscillator is zero; since it simultaneously generates the excitation field and detects the spin-induced perturbation, no T/R switching or resonator ring-down occurs. The detector therefore remains continuously operational throughout the experiment.

The deterministic transient is the response of the PLL to the frequency step occurring at the start and end of the excitation pulse. This response consists of an initial large-signal settling phase, whose duration depends on the applied frequency step, followed by the linear settling of the PLL. Once the PLL has entered its linear operating regime, the remaining transient is governed entirely by the poles and zeros of the closed-loop transfer function. Consequently, for a given frequency step, both the large-signal response and the subsequent linear settling are highly reproducible. Furthermore, modern PLL architectures provide circuit techniques that maintain lock even for comparatively large frequency steps, thereby avoiding excessive nonlinear recovery.

The receiver chain’s dynamic range dictates how these dynamics manifest in practice. Because the PLL demodulates the MR signal to baseband, subsequent signal-conditioning electronics process a low-bandwidth MR signal (typically a few tens of kilohertz) alongside the low-frequency deterministic transient (extending to a few megahertz). These subsequent stages introduce negligible additional settling artifacts, and the overall settling dynamics are dominated almost entirely by the PLL itself. In the current prototype, this transient is removed in the digital domain after acquisition, which minimizes hardware complexity, but when weak MR signals require a large overall gain, the transient can saturate the amplification chain, extending the interval over which digital subtraction cannot recover the signal. Alternatively, the same deterministic transient can be removed in the analog domain before high-gain amplification using a low-noise, high-speed digital-to-analog converter and a differential subtraction stage. This allows the analog gain to be optimized for the expected MR signal alone, since the LNA and the subsequent signal-conditioning stages process only the small residual subtraction error in addition to the desired MR signal. This strategy is conceptually similar to resonator ring-down cancelation in conventional transceiver architectures. However, unlike conventional MR, where the transient is shaped by the complex, coupled, and often nonlinear dynamics of the resonator, power amplifier, T/R switch, and protection circuitry, the transient here is defined almost exclusively by the simpler, well-behaved dynamics of the oscillator–PLL system. The lower number of components, along with an intrinsic shift of the signal to baseband frequencies, makes deterministic analog cancelation more practically viable.

While a controlled synthetic signal experiment provides a clean proof of principle for this transient-cancelation approach (see fig. S3), implementing that this procedure on a real spin system reveals the practical challenges of the technique. In a phase-cycled Hahn echo experiment performed on our low-field system, the deterministic transient was removed by subtracting a reference acquisition recorded at a slightly offset static magnetic field. Although this subtraction successfully recovers the signal, the cancelation is less complete than in the idealized synthetic case, leaving a residual transient. This vulnerability to parameter drift highlights a fundamental distinction between closed-loop phase coherence and transient trajectories: Phase coherence is a steady-state, closed-loop property that is largely insensitive to analog component drift because the PLL actively suppresses deviations against an external reference; the transient trajectory, by contrast, depends on the instantaneous, temperature-dependent values of loop parameters at the moment of the frequency step, most notably the VCO tuning sensitivity, which is intrinsically temperature-dependent through its semiconductor varactor characteristics, alongside contributions from off-chip components. While the oscillator chip itself is temperature stabilized to ∼0.01°C, residual thermal drift of off-chip loop components between the active and reference acquisitions dominates the observed residual transient. The impact of this residual depends critically on both the MR signal amplitude and the sample *T*_2_^*^. Achieving reliable, long-term subtraction therefore requires a comprehensive thermally stabilized system. In our current prototype, accommodating this stabilization inside the small-bore magnet is restricted by space, as moving the coil would push it into regions of lower-field homogeneity, substantially degrading the SNR. Future implementations using larger-bore magnets or integrated thermal stabilization within homogeneous-field regions will be essential to fully exploit this transient cancelation approach in demanding real-spin experiments.

Beyond transient and dynamic range considerations, the achievable *B*_1_ field in the current implementation is relatively modest, resulting in a π/2 pulse length of ∼100 μs. These *B*_1_ field strengths are fully sufficient for common spectroscopy, relaxometry, and imaging but limit access to very short-lived spin species and reflect a broader constraint shared by many on-chip and integrated MR detectors, where voltage headroom and power handling in conventional CMOS technologies restrict the attainable drive strength for large sensitive volumes. Recent advances in wide-bandgap semiconductor technologies, such as GaN-based RF electronics, offer a clear pathway toward substantially higher *B*_1_ fields comparable to those of commercial systems. While these approaches will increase power dissipation and cooling requirements, these challenges are primarily engineering in nature and appear tractable within the constraints of compact probe designs.

Another important practical consideration is the spin sensitivity of the present 477-MHz system prototype, which is approximately three orders of magnitude below that of a common 400-MHz NMR system with inductive detection (see section S3 for a detailed analysis). However, only a minor fraction of this gap is intrinsic to the frequency variation detection principle itself; assuming identical resonators, oscillator-based detection exhibits the same fundamental SNR scaling as conventional inductive detection, carrying only a fixed intrinsic noise figure penalty of ∼3 dB to sustain the limit cycle against active transistor noise. Instead, the observed sensitivity gap can be quantitatively decomposed into two dominant, implementation-specific factors. First, the measured root mean square noise of 11.4 μV (in a 100-kHz bandwidth) is roughly one order of magnitude higher than the theoretically predicted thermal Johnson noise limit of the oscillator (1.2 μV), an excess consistent with previous integrated LC oscillator benchmarks attributing these deviations to active device noise and addressable experimental issues. Second, the remaining two orders of magnitude in the gap stem directly from the coil geometry, specifically, a factor of 20 to 30 reduction in the magnetic field per unit current (*B*_u_) and the high resistance (low-quality factor) of our PCB-based segmented 13-mm single-turn coil compared to the multiturn 5-mm coil assumed for the conventional detector. The presented segmented PCB coil was intended to demonstrate the scalability of the segmented-coil oscillator array concept to NMR frequencies rather than to maximize filling factor and performance. This sensitivity deficit is not a fundamental limitation of oscillator-based MR and is expected to narrow substantially through the coil and *B*_1_ optimization pathways outlined above.

Looking forward, several steps are required to translate these findings into broader MR applications. Improved thermal management would facilitate deterministic transient subtraction in real-spin experiments. Extension of the architecture to higher frequencies and faster spin dynamics will require further optimization of loop bandwidth and *B*_1_ strength, but the underlying detection principle remains unchanged. Last, the combination of continuous acquisition and scalable oscillator arrays opens opportunities for pulse sequences and measurement strategies that directly exploit uninterrupted access to spin dynamics, rather than compensating for dead time with complex measures.

In summary, this work shows that oscillator-based MR detection, embedded into a fast PLL, can combine detection of Rabi oscillations during the excitation pulse, phase-coherent multipulse operation, and a deterministic, in-principle fully removable dead time transient within a compact and scalable MR detector architecture. Rather than eliminating physical limitations entirely, oscillator-based detection trades traditional receiver recovery and resonator ring-down for addressable challenges in dynamic range and deterministic transient cancelation, providing a practical framework to capture fast spin dynamics.

## MATERIALS AND METHODS

### High-field VCO-based NMR setup

The VCO array chip was fabricated in the ams-OSRAM 0.35-μm HV CMOS technology. The chip contains 10 injection-locked VCO cores, each comprising a cross-coupled transistor pair, a diode varactor, and a current mirror for biasing, similar to the previous design (*20*). The fabricated chip has a measured VCO tuning sensitivity of 4.8 MHz/V at the operating point used here, a measured open-loop phase noise of −97 dBc/Hz at 100 kHz, and a power consumption of 0.8 W from a 4.5-V supply. The planar NMR coil is implemented on a custom PCB functioning as an NMR probe head, containing, apart from the VCO chip and coil segments, only passive components, commercial buffers for signal conditioning, and a custom liquid sample holder (see Fig. 2), three-dimensional (3D) printed using Formlabs Form 3 printer and Clear v4 resin.

The PLL is implemented on a separate PCB outside the magnet using Analog Devices HMC439 as phase detector and charge pump and an active loop filter. The PLL has a measured bandwidth of ∼2 MHz. The PLL reference frequency was generated using a Rohde & Schwarz SMA100B signal generator. Tuning voltage transients were recorded interchangeably using a Rohde & Schwarz MXO44 oscilloscope and a Zürich Instruments MFLI Digitizer.

### High-field NMR measurements

The high-field (477-MHz) NMR measurements were performed in a sweepable superconducting magnet with a maximum field strength of 11.74 T from Cryogenics Ltd., with an accessible bore diameter of 25 mm. Frequency hopping was performed by generating a square signal using an output channel of an oscilloscope, which was used for frequency modulation of the signal generator and for triggering the data acquisition. Pulse lengths were calibrated using a nutation plot performed on distilled water (see fig. S2). FT-NMR was performed on undiluted acetic acid (Sigma-Aldrich). Rabi/FID and SSFP measurements were performed on distilled water. The repetition time for the experiments was 7 s.

### Low-field NMR setup

The VCO core chip is the same as previously reported in (*20*). In short, it was fabricated in the ams-OSRAM 0.35-μm HV CMOS technology and contains a single cross-coupled transistor pair, a diode varactor, a pair of switchable capacitors, and a current mirror for biasing. A two-layer (six turns per layer) solenoid coil with an inner diameter of 3 mm and a length of 5 mm, wound with 0.55-mm copper wire, was used as the tank inductor. A new PCB was designed for the present experiments, containing additional components, most notably a discrete PLL comprising the same active components as in the high-field implementation. The PLL reference, as well as the timing signals for data acquisition, was generated using the Keysight M8190A arbitrary waveform generator controlled by a custom script written in Python. The tuning voltage data were recorded using the Rohde & Schwarz MXO44 oscilloscope.

### Low-field NMR measurements

The low-field (17-MHz) NMR measurements were performed in a Bruker EPR electromagnet. Pulse lengths were calibrated using a nutation plot (see fig. S3). All measurements were performed on distilled water. A repetition time of 4 s was used, resulting in only negligible saturation (signal intensity of >97% of that obtained with a repetition time of 7 s).

## Supplementary Materials

### This PDF file includes:

Supplementary Text Figs. S1 to S4 Tables S1 and S2 References
