# Supplementary material for: Concurrent excitation and detection in magnetic resonance using voltage-controlled oscillators
Source: Sci Adv. 2026 Sep 16;12(38):eaeg6581. doi: 10.1126/sciadv.aeg6581 (PMC13580536; doi:10.1126/sciadv.aeg6581)
Supplement: Supplementary file 1 [file sciadv.aeg6581_sm.pdf]

Science Advances
AAAS

Supplementary Materials for

Concurrent excitation and detection in magnetic resonance using voltage-controlled oscillators

Zhibin Zhao *et al.*

Corresponding author: Michal Kern, michal.kern@iis.uni-stuttgart.de

Sci. Adv. **12**, eaeg6581 (2026)
DOI: 10.1126/sciadv.aeg6581

This PDF file includes:

Supplementary Text
Figs. S1 to S4
Tables S1 and S2
References

Supplementary Materials

S1. High-field system implementation details

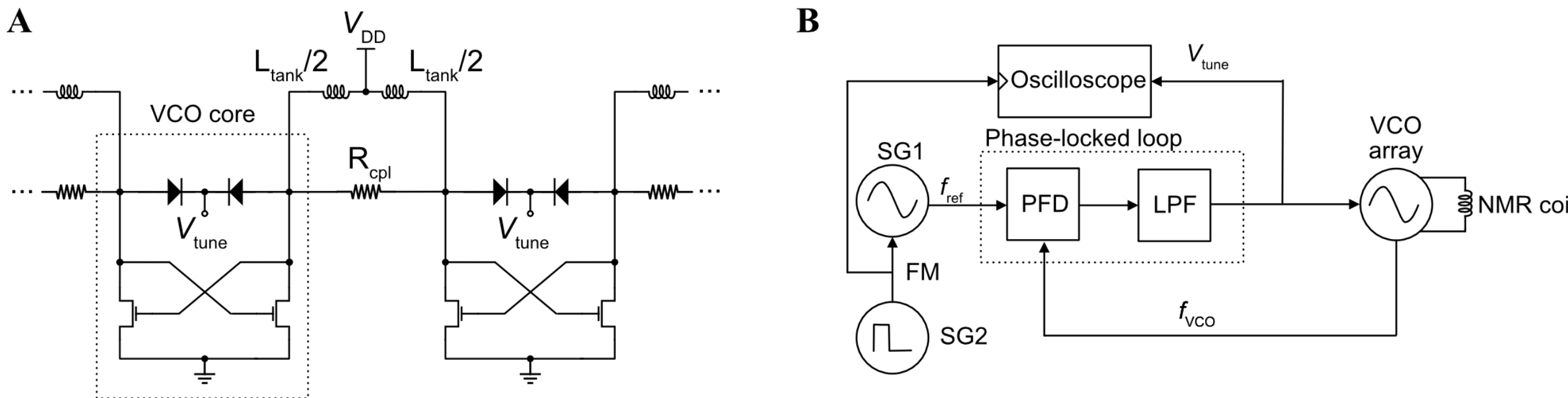

Fig. S1 (A) Simplified schematic of two injection-locked VCO cores. Each core uses a cross-coupled transistor pair with a split tank inductor (L_{tank}) and a diode varactor with tuning voltage (V_{tune}). Adjacent cores are mutually coupled through on-chip resistors R_{cpl}. (B) Block diagram of the VCO controlled by an off-chip PLL: a phase-frequency detector (PFD) compares a reference frequency f_{ref} from a signal generator (SG1) and the instantaneous frequency of the VCO array f_{VCO}. The PFD output is filtered by a low-pass filter (LPF) to produce the VCO control voltage V_{tune}. V_{tune} is simultaneously measured using an oscilloscope. A square signal from a second signal generator (SG2) is used to generate frequency modulation (FM) to perform pulsed measurements and trigger the data acquisition.

Each VCO core (Fig. S1A) consists of a cross-coupled differential transistor pair with a center-tapped tank inductor split into two halves (L_{tank}) and varactor diodes controlled by V_{tune}. The ten cores are arranged in a 4–4–2 configuration across three groups, each driving an off-chip PCB segmented loop inductor. Inter-core coupling through 5 Ω resistors (R_{cpl}) enables injection locking, forcing all cores to a common frequency and phase despite minor inductance mismatches among the coil segments. Coil inductances were optimized using a genetic algorithm in CST Studio to keep the deterministic mismatch below 5%, ensuring reliable synchronization across the full array.

During operation, a PLL (Fig. S1B) locks the VCO array frequency to an external reference (SG1). The phase-frequency detector output, after low-pass filtering, generates V_{tune}, which is simultaneously recorded on an oscilloscope. Pulsed NMR measurements are performed by applying a square-wave FM signal from a second generator (SG2), which also serves as the acquisition trigger.

S2. Pulse length calibration

S2.1. High-field system

A nutation experiment was performed by incrementing the excitation pulse width t_p while keeping other drive conditions constant. For each pulse width t_p, the magnitude of the FID peak in its Fourier transform was extracted. The resulting dataset is shown in Fig. S.
In the absence of inhomogeneities, the transverse magnetization follows

$$M_\perp \propto \sin(\omega_R t_p),$$
$$\omega_R = \gamma B_1,$$

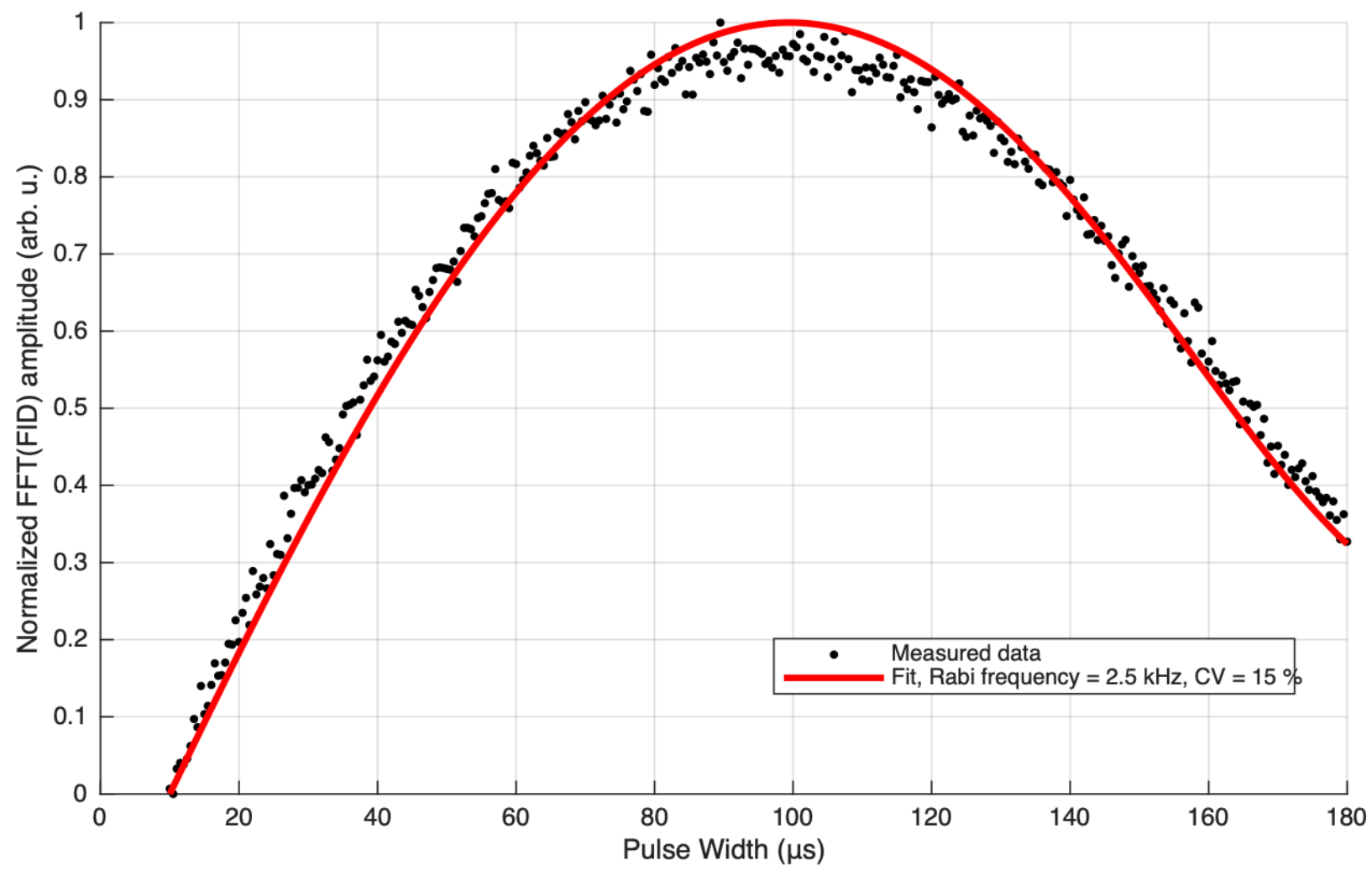

Fig. S2 Pulse length calibration. Nutation plot showing the FID amplitude after Fourier transform as a function of pulse length. The fitted Rabi frequency $\omega_R/2\pi$ = 2.5 kHz with a coefficient of variation CV = 15% (inhomogeneity) across the sensitive volume. The resulting π /2-pulse length is approximately 100 µs.

Where ω_R is the Rabi frequency in angular units and γ is the proton gyromagnetic ratio. To account for spatial variations of B_1 across the sensitive volume, the measured data was fitted with an ensemble-averaged sinusoidal response over a log-normal distribution of Rabi frequencies f

$$y(t_p) = \int_0^\infty |\sin(2\pi f t_p)| p_{LN} df,$$

$$p_{LN} = \frac{1}{f\sigma\sqrt{2\pi}} \exp\left(-\frac{(\ln f - \mu)^2}{2\sigma^2}\right), \qquad (S1)$$

Where $\sigma = \sqrt{\ln(1+r^2)}$, $\mu = \ln f_0 - \frac{1}{2}\sigma^2$, $r = \frac{\sigma_f}{f_0}$, with mean Rabi frequency $f_0 = \omega_R/2\pi$, standard deviation σ_f and coefficient of variation (CV) r.

S2.2. Low-field system

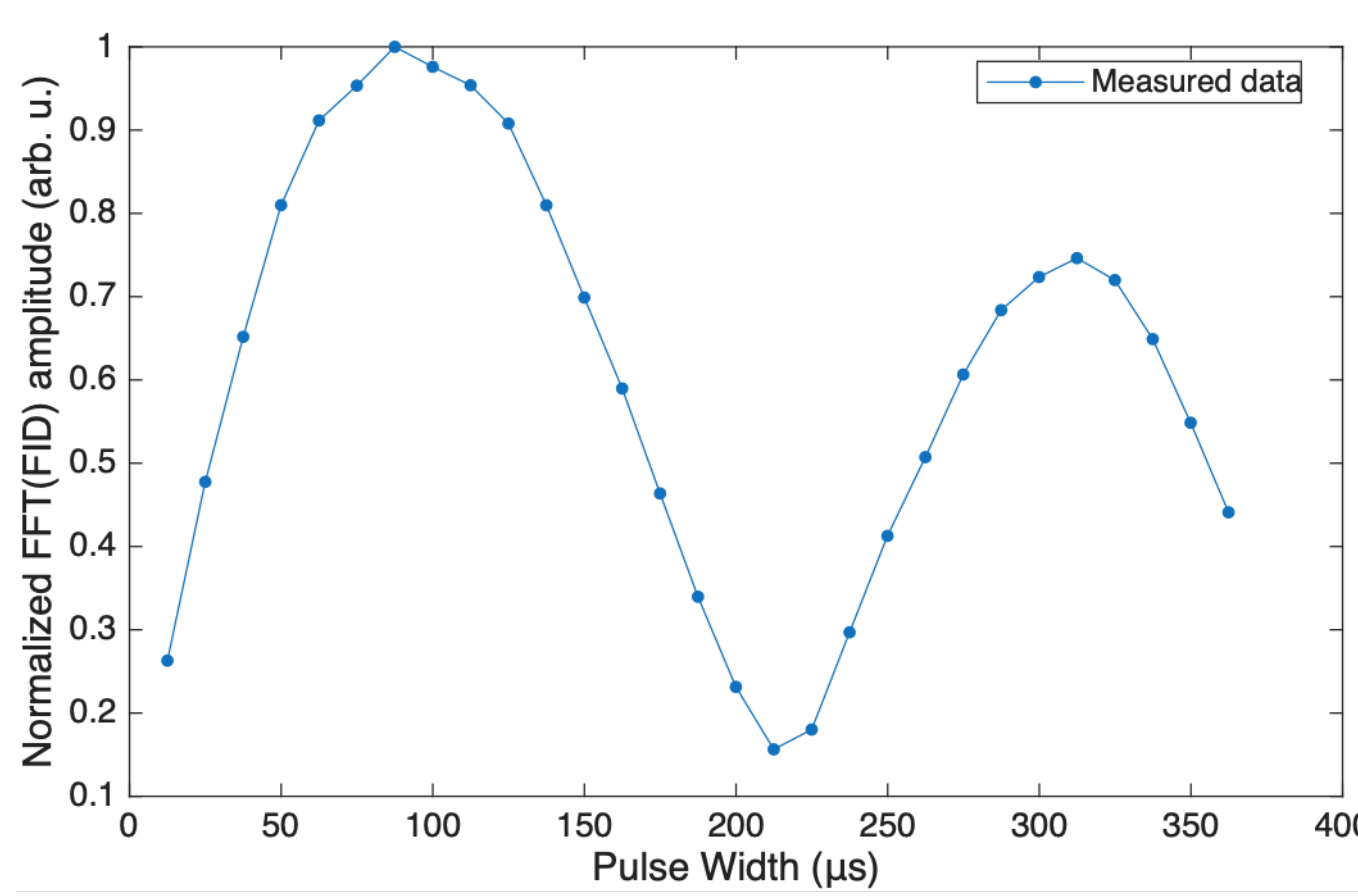

Fig. S3 Pulse length calibration of the low-field NMR system. Nutation plot showing the FID amplitude after Fourier transform as a function of pulse length. The optima π /2-pulse length is approximately 100 µs.

S3. Signal, noise, and spin sensitivity: theory and comparison to conventional NMR

S3.1. Signal and noise in oscillator-based magnetic resonance

The B_0^3 dependence of the oscillator frequency deviation reported by Hassan *et al.* (*26*) is predicted by a perturbation model and originates from two independent physical mechanisms: the magnetic resonance process itself and the response of the autonomous oscillator.

The first contribution is identical to that of conventional inductive detection. As discussed by Hould and Richards (*29*), the precessing spin magnetization induces an electromotive force in the resonator according to Faraday's law,

$$v_{\mathrm{emf}}(t) = -\frac{\mathrm{d}}{\mathrm{d}t}\int_{Vs} \mathbf{B}_u(\mathbf{r}) \cdot \mathbf{M}_s(\mathbf{r},t)\mathrm{d}V\,. \tag{S2}$$

Since the equilibrium magnetization scales as $M_0 \propto B_0$ and the time derivative introduces the Larmor frequency $\omega_L \propto B_0$, the induced emf exhibits the familiar $v_{\mathrm{emf}} \propto B_0^2$ dependence known from conventional inductive detection.

The second contribution originates from the oscillator itself. The spin-induced emf acts as a small perturbing force on the stable oscillator limit cycle. Such a perturbation can be decomposed into amplitude and phase components. According to Chu *et al.* (*30*), the amplitude perturbation is governed by

$$\delta\dot{A}(t) = -\underbrace{(\alpha_{\mathrm{od}} - 1)\left(\frac{\omega_{\mathrm{osc}}}{Q_{\mathrm{coil}}}\right)}_{\equiv\omega_p}\delta A - \omega_{\mathrm{osc}} \cdot v_{\mathrm{emf}}(t) \cdot \sin(\omega_{\mathrm{osc}}t) \tag{S3}$$

and is suppressed by the oscillator's intrinsic amplitude control mechanism, acting as a first-order low-pass filter for perturbations such as $v_{\mathrm{emf}}(t)$ outside the bandwidth of the above ODE, i.e. outside $\omega_p = (\alpha_{\mathrm{od}} - 1)\left(\frac{\omega_{\mathrm{osc}}}{Q_{\mathrm{coil}}}\right)$. $\alpha_{\mathrm{od}} = G_{\mathrm{m}}/G_{\mathrm{t}}$ is the overdrive parameter, which indicates by how much the negative transconductance from the cross-coupled transistor pair overcompensate the coil loss and $G_{\mathrm{m}}, G_{\mathrm{t}}$ are the transistor gate and LC tank transconductance, respectively. Also, importantly, within the bandwidth of the above ODE, the sensitivity that translates an emf into amplitude fluctuations is given by $\frac{Q_{\mathrm{coil}}}{\alpha_{\mathrm{od}}-1}$, i.e. independent of ω_{osc}.

In contrast, the oscillator phase possesses no corresponding restoration mechanism because the limit cycle is invariant under time translation. Consequently, perturbations such as the emf predominantly accumulate as phase shifts rather than amplitude changes. As a result, the perturbing emf produces a phase shift that is, to first order, approximately independent of the oscillation frequency. As was shown by Anders *et al.* in (*31*), the corresponding differential equation for the phase perturbations resulting from an induced emf in the coil is given by:

$$\delta\dot{\varphi} = -\beta_1\delta A - \frac{\omega_{\mathrm{osc}}}{A_0} \cdot v_{\mathrm{emf}}(t) \cdot \cos(\omega_{\mathrm{osc}}t) \approx -\frac{\omega_{\mathrm{osc}}}{A_0} \cdot v_{\mathrm{emf}}(t) \cdot \cos(\omega_{\mathrm{osc}}t)\,. \tag{S4}$$

Interestingly, due to the relation between the instantaneous phase φ and the instantaneous frequency ω_i according to $\omega_i = \frac{d\varphi_i}{dt}$, using the relation between instantaneous phase and instantaneous frequency transforms the differential equation for the phase fluctuations into the following algebraic expression for the frequency deviation:

$$\delta\omega \approx -\frac{\omega_{\text{osc}}}{A_0} \cdot v_{\text{emf}}(t) \cdot \cos(\omega_{\text{osc}} t)\,. \tag{S5}$$

According to (S5), the absolute frequency deviation produced by an emf v_{emf} induced in the coil grows proportionally to the oscillation frequency ω_{osc}.

Equivalently, the oscillator exhibits an approximately constant normalized frequency sensitivity, i.e.,

$$\frac{\delta\omega}{\omega_{\text{osc}}} \approx -\frac{1}{A_0} \cdot v_{\text{emf}}(t) \cdot \cos(\omega_{\text{osc}} t) \tag{S6}$$

for a given perturbing emf. The corresponding phase fluctuations are given by

$$\delta\varphi \approx -\frac{1}{A_0} \cdot v_{\text{emf}}(t) \cdot \cos(\omega_{\text{osc}} t) \tag{S7}$$

And are independent of the oscillation frequency ω_{osc}.

In summary, the emf induced in the tank coil shows the conventional B_0^2 scaling. The oscillator transduces this emf into both amplitude and phase perturbations through sensitivity functions that are essentially independent of the carrier frequency. Only the conversion from phase to instantaneous frequency introduces an additional factor of ω_{osc}, resulting in the observed B_0^3 scaling of the frequency signal.

Interestingly, a detailed noise analysis of the cross-coupled pair LC tank oscillator (*31–33*), reveals that the sensitivity function between the coil noise and the amplitude noise are given by equations (S3) and (S4), when the deterministic amplitude A_0 is replaced by its smoothly averaged version A_{sm} (*33*) and the emf is replaced by a random process that models the coil noise according to:

$$\delta\dot{A}(t) = -\underbrace{(\alpha_{\text{od}} - 1)\left(\frac{\omega_{\text{osc}}}{Q_{\text{coil}}}\right)}_{\equiv\omega_p} \delta A - \omega_{\text{osc}} \cdot v_{\text{noise,R}}(t) \cdot \sin(\omega_{\text{osc}} t) \tag{S8}$$

$$\delta\dot{\varphi} = -\beta_1 \delta A - \frac{\omega_{\text{osc}}}{A_{\text{sm}}} \cdot v_{\text{emf}}(t) \cdot \cos(\omega_{\text{osc}} t) \approx -\frac{\omega_{\text{osc}}}{A_{\text{sm}}} \cdot v_{\text{noise,R}}(t) \cdot \cos(\omega_{\text{osc}} t) \tag{S9}$$

The resulting power spectral densities of the amplitude and the frequency noise are given by (*31*, *31*, *33*):

$$S_{\delta A \delta}(\omega) = \frac{1}{(\alpha_{\text{od}} - 1)^2} \cdot Q_{\text{coil}}^2 \cdot kTR_{\text{coil}} \cdot \frac{1}{\left(1 + \frac{\omega}{\omega_c}\right)^2} \tag{S10}$$

$$S_{\delta\omega\delta\omega}(\omega) = kT \left(\frac{\omega_{\mathrm{osc}}}{A_0}\right)^2 R_{\mathrm{coil}} \tag{S11}$$

Moreover, it can be shown that the total noise originating from the cross-coupled transistor pair and the coil resistance can be approximated by:

$$S_{\delta A\delta}(\omega) = \frac{1 + \alpha_{\mathrm{od}}\gamma_{\mathrm{nd}}}{(\alpha_{\mathrm{od}} - 1)^2} \cdot Q_{\mathrm{coil}}^2 \cdot kTR_{\mathrm{coil}} \cdot \frac{1}{\left(1 + \frac{\omega}{\omega_c}\right)^2} \tag{S12}$$

$$S_{\delta\omega\delta\omega}(\omega) = kT \left(\frac{\omega_{\mathrm{osc}}}{A_0}\right)^2 (1 + \alpha_{\mathrm{od}}\gamma_{\mathrm{nd}})R_{\mathrm{coil}}, \tag{S13}$$

where ω is the offset frequency from the oscillation frequency. $\gamma_{\mathrm{nd}} \approx 1$ is the so-called gate excess noise factor of the transistors.

Consequently, the resulting SNRs in the amplitude (white noise region) and the frequency (white noise region) signals show the following dependencies on the oscillation frequency ω_{osc}:

$$\mathrm{SNR}_{\delta A} \propto \frac{\frac{Q_{\mathrm{coil}}}{\alpha_{\mathrm{od}} - 1} \cdot \hat{v}_{\mathrm{emf}}}{\sqrt{\frac{1 + \alpha_{\mathrm{od}}\gamma_{\mathrm{nd}}}{(\alpha_{\mathrm{od}} - 1)^2} \cdot Q_{\mathrm{coil}}^2 \cdot kTR_{\mathrm{coil}}}} \propto B_0^2 \tag{S14}$$

$$\mathrm{SNR}_{\delta\omega} \propto \frac{\frac{\omega_{\mathrm{osc}}}{A_0} \cdot \hat{v}_{\mathrm{emf}}}{\sqrt{kT \left(\frac{\omega_{\mathrm{osc}}}{A_0}\right)^2 (1 + \alpha_{\mathrm{od}}\gamma_{\mathrm{nd}})R_{\mathrm{coil}}}} \propto \frac{B_0 \cdot B_0^2}{B_0} = B_0^2 \tag{S15}$$

Interestingly, according to (S14) and (S15), both the MR signal encoded in the oscillation amplitude and the one encoded in the oscillation frequency are displaying the conventional B_0^2-scaling in the achievable SNR.

Equations (S12) and (S13) show that the finite oscillator overdrive and the transistors' excess noise degrade the frequency-noise floor, and hence the achievable SNR, relative to the coil-thermal-noise-only limit by a factor $\sqrt{(1 + \alpha_{\mathrm{od}}\gamma_{\mathrm{nd}})}$. For near-optimal, still realistic transistor bias points $\alpha_{\mathrm{od}} = 1.5$, $\gamma_{\mathrm{nd}} = 0.65$ (see (*34*)) this gives $\sqrt{(1 + 1.5 \cdot 0.65)} \approx \sqrt{2} \approx 1.4$, corresponding to an SNR degradation of $20 \log_{10} 1.4 = 3$ dB relative to conventional, thermally-limited inductive detection using the same coil. This is worse than achievable with a dedicated low-noise NMR preamplifier, but is the price paid for the concurrent, dead-time-free detection discussed in the main text, and the penalty becomes proportionally less important as the target detection frequency moves toward the EPR regime.

S3.2. Calculation of the expected signal amplitude in presented high-field experiments

According to the discussion in Section S3.1 and (*26*), the spin-induced modulation of the VCO output frequency is given by:

$$\varDelta\omega_{\text{osc,spin}}(t) \approx \frac{\omega_{\text{osc},0}}{A_{\text{osc},0}} \cos(\omega_{\text{osc},0} t) \cdot \frac{\mathrm{d}}{\mathrm{d}t} \int_{V_s} \mathbf{B}_u \cdot \mathbf{M}_{\text{spin}} \, \mathrm{d}V, \qquad \text{(S16)}$$

where $\omega_{\text{osc},0}$ and $A_{\text{osc},0}$ are the free-running oscillation frequency and amplitude, $\mathbf{B}_u$ is the unitary magnetic field of the tank coil, and $\mathbf{M}_{\text{spin}}$ is the sample magnetization. This expression follows the same nonlinear-perturbation formalism used for the oscillator phase-noise derivation by Anders (*35*) and Section S3.1. Modeling $\mathbf{M}_{\text{spin}}$ according to $\mathbf{M}_{\text{spin}} = \widehat{\mathbf{M}}_{\text{spin}} \cdot \exp(-\frac{t}{T_2^*}) \cdot \sin(\omega_{\text{L}} t)$, ignoring the term at $\omega_{\text{osc},0} + \omega_{\text{L}}$, and assuming $\omega_{\text{L}} \gg \frac{1}{T_2^*}$ (valid for our experiment), we obtain:

$$\varDelta\omega_{\text{osc,spin}}(t) \approx \frac{\omega_{\text{osc},0} \cdot \omega_{\text{L}}}{2 \cdot A_{\text{osc},0}} \cos((\omega_{\text{osc},0} - \omega_L) \cdot t) \cdot \exp\left(-\frac{t}{T_2^*}\right) \cdot \int_{V_s} \mathbf{B}_u \cdot \widehat{\mathbf{M}}_{\text{spin}} \, \mathrm{d}V, \qquad \text{(S17)}$$

Since for a PLL in the locked state, within its bandwidth, the PLL forces the VCO frequency to follow the PLL's reference frequency. Therefore, in the presence of a spin-induced frequency deviation according to equation (S16), and, for simplicity, introducing $\omega_{\text{osc},0} = \omega_{\text{L}} + \Delta\omega_{\text{off}}$ the PLL produces a counteracting signal according to

$$\Delta V_{\text{tune}} = -\frac{\varDelta\omega_{\text{spin}}(t)}{K_{\text{VCO}}} \approx$$
$$\approx -\frac{1}{K_{\text{VCO}}} \cdot \frac{\omega_{\text{osc},0}}{A_{\text{osc},0}} \cdot \frac{\omega_{\text{L}}}{2} \cdot \cos(\Delta\omega_{\text{off}} t) \cdot \exp\left(-\frac{t}{T_2^*}\right) \cdot \int_{V_s} \mathbf{B}_u \cdot \widehat{\mathbf{M}}_{\text{spin}} \, \mathrm{d}V, \qquad \text{(S18)}$$

where K_{VCO} is the VCO tuning sensitivity, also referred to as the VCO gain, in ((rad/s)/V).

Assuming a uniform B_u-field according to $\mathbf{B}_u = B_{ux}\mathbf{e}_x$, we can further simplify equation , yielding

$$\Delta V_{\text{tune}} = -\frac{1}{K_{\text{VCO}}} \cdot \frac{\omega_{\text{osc},0} \cdot \omega_L}{2 \cdot A_{\text{osc},0}} \cdot \cos(\Delta\omega_{\text{off}} t) \cdot \exp\left(-\frac{t}{T_2^*}\right) \cdot \int_{V_s} B_{ux} \cdot \widehat{M}_{xy,0} \, \mathrm{d}V, \qquad \text{(S19)}$$

where $M_{xy,0}$ is the magnetization tipped into the transverse plane and T_2^* is the observed decay time. The peak value of the signal described by (S18) is given by

$$|\Delta V_{\text{tune}}|_{\text{peak}} = \frac{1}{K_{\text{VCO}}} \cdot \frac{\omega_{\text{osc},0} \cdot \omega_{\text{L}}}{2 \cdot A_{\text{osc},0}} \cdot \int_{V_s} B_{ux} \cdot \widehat{M}_{xy,0} \, \mathrm{d}V \approx$$
$$\approx \frac{1}{K_{\text{VCO}}} \cdot \frac{\omega_{\text{osc},0} \cdot \omega_{\text{L}}}{2 \cdot A_{\text{osc},0}} B_{ux} \cdot M_{xy,0} \cdot V_s, . \qquad \text{(S20)}$$

For a spin-$1/2$ ensemble at proton concentration c and temperature T,

$$M_0 = \frac{cN_A\gamma^2\hbar^2}{4kT}B_0, \qquad M_{xy,0} = M_0\sin\theta, \tag{S21}$$

where γ is the proton gyromagnetic ratio, N_A is Avogadro's number, k_B is Boltzmann's constant, B_0 is the static field, and θ is the excitation flip angle.

$\mathbf{B}_u(\mathbf{r}) = \mathbf{B}(\mathbf{r})/I$ is the coil field per unit drive current, related to the transmit field by reciprocity. With B_1 measured at the sample position via the Rabi nutation calibration and $\hat{I}$ the corresponding tank current amplitude,

$$B_u = \frac{B_1}{\hat{I}}. \tag{S22}$$

The manuscript's quoted sensitive volume (575 µL) corresponds to a hemisphere of radius equal to the coil radius a:

$$V_{\text{hemi}} = \frac{2}{3}\pi a^3. \tag{S23}$$

Because the sample was offset by a distance z from the coil surface, a cylinder of height z nearest the coil is unoccupied by sample and is subtracted:

$$V_{\text{cyl}}(z) = \pi a^2 \text{z}, \qquad V_s = V_{\text{hemi}} - V_{\text{cyl}}(z). \tag{S24}$$

Table S1 Parameters used for the calculation of expected FID peak amplitude for HF VCO-based NMR.

Symbol	Description	Value
B_0	Static field	11.74 T
T	Sample temperature	300 K
c	Proton concentration (acetic acid, CH_3 protons)	52,410 $\frac{\text{mol}}{\text{m}^3}$
a	Coil radius	6.5 mm
z	Sample offset from coil surface	1 mm
θ	Excitation flip angle	$\pi/2$
B_1	On-resonance field at sample (Rabi calibration)	59 µT
$\omega_{\text{osc},0}/2\pi$	Carrier frequency	477 MHz
$A_{\text{osc},0}$	Oscillation amplitude	3 V
$\hat{I}$	Tank current	30 mA
K_{vco}	VCO tuning sensitivity	$2\pi \cdot$ 4.8 Mrad/s/V

With the parameters specified in Table S1, the resulting expected FID amplitude is 1.2 mV, compared to the measured ~1.5 mV. There are number of uncertainties and approximations in the calculation: the actual sensitive volume may exceed the hemispherical estimate due to the fringing field, and, more importantly, the tank current is difficult to both measure and simulate

due to the complex nature of the PCB-based segmented coil and the associated parasitics. Within these approximations, the theoretical expression captures the correct order of magnitude.

S3.3. Thermally limited oscillator frequency noise

The thermally limited frequency noise power spectral density of an LC oscillator can be expressed as (*36*):

$$S_{\delta\omega\delta\omega}(f) = kT \cdot (1 + \alpha_{\mathrm{od}}\gamma_{\mathrm{nd}}) \cdot \left(\frac{\omega_{\mathrm{osc},0}}{A_{\mathrm{osc}}}\right)^2 R_{\mathrm{coil}}, \tag{S25}$$

With R_{coil} being the coil resistance, $\alpha_{\mathrm{od}} = G_{\mathrm{m}}/G_{\mathrm{t}}$ the overdrive parameter, which indidatces by how much the negative transconductance from the cross-coupled transistor pair overcompensate 26the coil loss, and $\gamma_{\mathrm{nd}} \approx 1$ is the so-called gate excess noise factor (G_{m}, G_{t} are the transistor gate and LC tank transconductance, respectively). The resulting rms frequency fluctuations over a bandwidth Δf are given by

$$\Delta\omega_{\mathrm{rms}} = \sqrt{S_{\delta\omega\delta\omega}(f) \cdot \Delta f} = \sqrt{kT \cdot (1 + \alpha_{\mathrm{od}}\gamma_{\mathrm{nd}}) \cdot R_{\mathrm{coil}} \cdot \Delta f} \cdot \left(\frac{\omega_{\mathrm{osc},0}}{A_{\mathrm{osc}}}\right), \tag{S27}$$

Evaluating this expression with $\alpha_{\mathrm{od}} = 2$, $\gamma_{\mathrm{nd}} = 1$ and dividing by K_{vco} yields a voltage noise floor of approximately 1.2 µV. The gap of approximately an order of magnitude to the measured value of 11.4 µV mirrors the one reported by Yalcin and Boero (*19*) and is commonly attributed to the additional noise introduced by the transistors in the oscillator circuit and additional instrumental noise. While our model does include the excess noise introduced by the oscillator circuit explicitly, there is an uncertainty in the exact value of the parameters during the presented experiments. An uncertainty in the estimation equivalent coil resistance similar to section S3.2 hast to be noted as well.

S3.4. Spin sensitivity of the HF VCO system

The spin sensitivity of a magnetic resonance experiment N_{min}, more precisely the limit of detection, is defined as the minimal number of spins detectable within 1 second of measurement time with a signal-to-noise ratio (SNR) of either 1 or 3 (constant *C*). This value can be calculated from an experiment as

$$N_{\mathrm{min}} = C \frac{N_{\mathrm{spins}}}{\mathrm{SNR}_{\mathrm{exp}} \sqrt{\Delta f}}, \tag{S28}$$

with N_{spins} being the number of spins measured. Setting *C* to 1 and using the experimental $\mathrm{SNR}_{\mathrm{exp}}$ of 1.5 mV / 11.4 µV ≈ 132 and recorded with a detection bandwidth of 100 kHz, we obtain $N_{\mathrm{min}} \approx 3.4 \times 10^{17} \frac{\mathrm{spins}}{\sqrt{\mathrm{Hz}}}$. The thermally limited case, taking the experimentally observed signal amplitude, would be an order of magnitude better, approximately $4.1 \times 10^{16} \frac{\mathrm{spins}}{\sqrt{\mathrm{Hz}}}$.

S3.5. Benchmark: conventional NMR probe

In order to gauge the presented system to conventional NMR spectrometer on a theoretical basis, we will compare the expected signal of a spectrometer operating at 400 MHz proton frequency with a 5 mm diameter solenoid probe. To estimate the achievable signal-to-noise ratio of the

solenoidal coil, we followed the reciprocity-based formalism of Hoult and Richards (*29*), which relates the NMR signal directly to the magnetic field produced by the coil under unit current, and the thermal noise directly to the coil's series resistance.

Due to the reciprocity principle, the emf induced in a coil by a precessing magnetization is

$$\xi = -\frac{\mathrm{d}}{\mathrm{d}t}\int_{V_s} \mathbf{B}_u \cdot \mathbf{M}_{\mathrm{spin}}\, \mathrm{d}V, \tag{S29}$$

with the variables as defined in Eq. (1). Following the same logic of section S1, the peak induced emf can be calculated as

$$\xi = B_{\mathrm{u,eff}}\, V_s\, M_0\, \omega_{\mathrm{L}}. \tag{S30}$$

The sample volume V_s is taken as the coil bore volume and the magnetization is calculated according to Eq. (6). Using the thin-loop approximation, the unitary field of a solenoid can be approximately calculated by

$$B_{\mathrm{u,eff}} = \frac{\mu_0 N}{2\sqrt{(l/2)^2 + r^2}}, \tag{S31}$$

where μ_0 is the permeability of vacuum N the number of turns, l the coil length and r it's radius.

The expected rms voltage noise, assuming it to come purely from the thermal noise of the coil, is given by

$$v_n = \sqrt{4kTR\Delta f}, \tag{S32}$$

with Δf being the measurement bandwidth and R the coil resistance. The AC resistance of the winding, including the proximity-effect correction factor ζ for a closely wound single-layer solenoid, is

$$R = \zeta\, \frac{l_{\mathrm{wire}}}{p_{\mathrm{wire}}} \sqrt{\frac{\mu_r \mu_0 \omega_{\mathrm{L}} \rho(T)}{2}}, \tag{S33}$$

where $l_{\mathrm{wire}} = 2\pi r N$ is the total wire length, $p_{\mathrm{wire}} = \pi d_{wire}$ its circumference, $\rho(T)$ the copper resistivity at temperature T and $\zeta \approx 3$ for a single-layer solenoid.

Table S2 Parameters used to calculate the expected SNR for a conventional NMR experiment.

Symbol	Parameter	Value
N	Turns	5
r	Coil radius	2.5 mm
l	Coil length	5 mm
d_w	Wire diameter	0.5 mm
f_0	Larmor frequency	400 MHz
B_0	Magnetic field	9.39 T

Using the parameters specified in Table S2 and acetic acid for comparison with the presented measurement, the expected peak FID amplitude is approximately 3.2 mV and a theoretical noise floor of approximately 34 nV in the same 100 kHz measurement bandwidth. The resulting SNR is approximately 90 000 and the corresponding limit of detection would be $\approx 1\ \times 10^{14}\,\frac{\mathrm{spins}}{\sqrt{\mathrm{Hz}}}$.

S3.6. Discussion of the difference between conventional and oscillator-based NMR

Comparing the spin sensitivity of the present VCO-based detector to that of a conventional inductively-detected NMR probe of similar sample volume and field, our system underperforms by ≈ 3 orders of magnitude. The gap can be decomposed as follows.

Section S3.1 shows that the minimal noise floor in frequency variation oscillator-based detection with optimal but realistic devices is degraded approximately 3 dB compared to thermal noise of the used coil only. Calculating the theoretical noise floor of our system with operating characteristics closer to the used device (Section S3.3) gives a number approximately one order of magnitude lower than the experimental noise, which we attribute to uncertainty in the exact operating characteristics and additional instrumental noise in the experiment. This one order of magnitude discrepancy has been reported multiple times in the oscillator-based EPR literature (*19*, *37*).

We attribute the remaining ≈2 orders of magnitude difference to the coil itself. A factor of 20 to 30 in the B_u can be estimated just from geometrical considerations, comparing the planar coil to a solenoid. The remaining discrepancy is most probably attributed to the coil resistance and correspondingly modest Q relative to a conventional NMR probe coil. This is consistent with the present PCB-based segmented coil having been designed for scalability and volume rather than for optimized filling factor or minimized loss. Taken together, these observations indicate that the orders-of-magnitude sensitivity gap between the present implementation and conventional NMR is set by identifiable, addressable engineering factors (coil geometry and resistance/Q first, and active-circuit noise second), rather than by a fundamental constraint of oscillator-based detection, consistent with the improvement pathway (larger B_1, optimized coil geometry) already outlined in the Discussion of the main text.

S4. True zero-dead-time-reconstruction

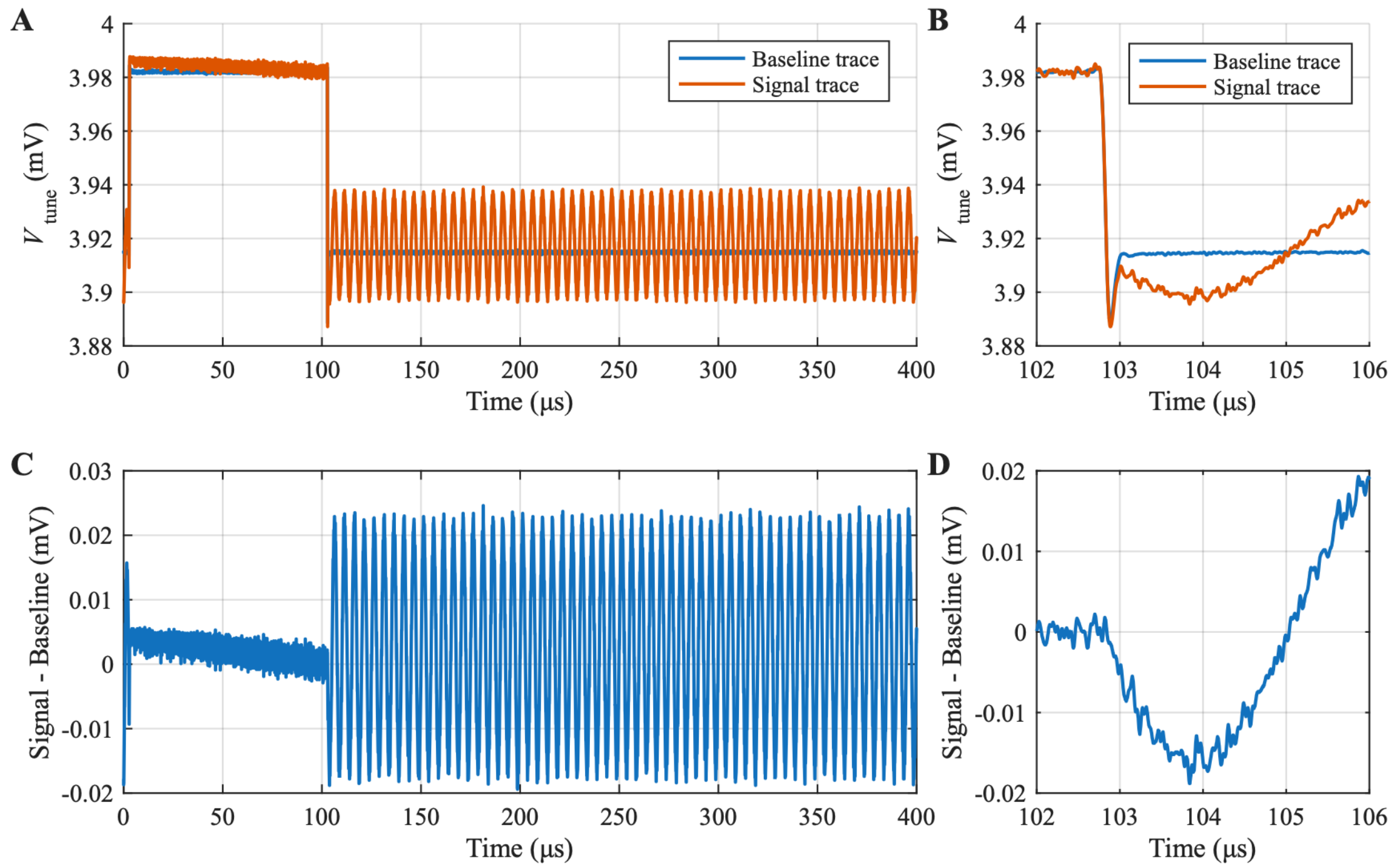

Fig. S4 Deterministic subtraction of the PLL transient demonstrated using a synthetic spin-like modulation. (A) Full V_{tune} trace showing the purely electrical PLL response (blue baseline; averaged over many repetitions) and a single-shot trace acquired with the injected synthetic signal (red). (B) Expanded view of the on-off resonance transition in A. (C) Difference trace obtained by subtracting the averaged baseline from the single-shot signal, isolating the injected modulation. (D) Expanded view of the on-off transition with background subtracted.

The measurements presented in the main text demonstrate that, under typical NMR conditions, the short PLL settling transient following a frequency hop does not limit practical operation. To determine whether this residual transient represents a fundamental limitation or whether it can be removed entirely, we performed a controlled electrical experiment that isolates the PLL response from spin-related effects.

In this experiment, the high-field VCO array was operated in the same PLL-referenced configuration as used for the NMR measurements, but outside of a magnetic field. A small sinusoidal modulation, injected externally through a wire loop, was used to emulate a spin-induced frequency deviation. Fig. S4 shows the resulting control voltage V_{tune} over a full excitation–detection cycle. The blue trace corresponds to the purely electrical PLL response recorded without injected modulation and averaged over many repetitions to obtain a high–signal-to-noise baseline. The red trace shows a single-shot measurement acquired with the injected synthetic signal, in which the deterministic PLL transient and the spin-like modulation are superimposed.

Fig. S4C shows the difference between the single-shot signal and the averaged baseline. After subtraction, the injected signal is recovered continuously across the frequency hop, with no missing data points or discontinuities. This demonstrates that the apparent dead time observed in the raw trace arises solely from the PLL's electrical response. The

slanted background during excitation arises from a small residual detuning of the injected modulation and the VCO frequency.

A magnified view of the on–off resonance transition in the raw data is shown in Fig. S4B, highlighting the high reproducibility of the PLL transient in the baseline and signal traces. Fig. S4D shows the corresponding zoom after subtraction, confirming that the transient is completely removed and that the synthetic signal is recovered without interruption through the transition.

To generate a synthetic spin signal for these zero-deadtime demonstration measurements, we mounted a single-loop wire coil above the VCO array using a 3D-printed fixture and injected a signal near the VCO-array frequency with a small detuning to simulate the spin signal using a Stanford Research SG396 signal generator.

REFERENCES

1. R. K. Harris, R. E. Wasylishen, Eds., *Encyclopedia of NMR* (Wiley, ed. 2, 2012).

2. W. G. Clark, Pulsed nuclear resonance apparatus. *Rev. Sci. Instrum.* **35**, 316–333 (1964).

3. D. J. Adduci, P. A. Hornung, D. R. Torgeson, Extremely fast recovery time receiver for pulsed NMR. *Rev. Sci. Instrum.* **47**, 1503–1505 (1976).

4. M. E. Stoll, Simple, fast recovery, low-noise receiver amplifier for pulsed NMR experiments. *Rev. Sci. Instrum.* **52**, 391–394 (1981).

5. P. Jezzard, C. J. Wiggins, T. A. Carpenter, L. D. Hall, J. A. Barnes, P. Jackson, N. J. Clayden, Demonstration of nuclear magnetic resonance imaging for void detection in carbon-fibre reinforced polymer composites, and comparison with ultrasound methods. *J. Mater. Sci.* **27**, 6365–6370 (1992).

6. R. Xie, L. Xiao, K.-J. Dunn, S. Fu, Determination of T2 distribution in the presence of significant internal field gradients. *J. Geophys. Eng.* **10**, 054008 (2013).

7. R. Enjilela, J. Guo, B. MacMillan, F. Marica, A. Afrough, B. Balcom, T1-T2* relaxation correlation measurements. *J. Magn. Reson.* **326**, 106961 (2021).

8. E. W. Hansen, Probing the "Dead-Time" in NMR by combining single pulse and solid echo experiments followed by a global model fit analysis. *Appl. Magn. Reson.* **55**, 477–496 (2024).

9. D. O. Brunner, L. Furrer, M. Weiger, W. Baumberger, T. Schmid, J. Reber, B. E. Dietrich, B. J. Wilm, R. Froidevaux, K. P. Pruessmann, Symmetrically biased T/R switches for NMR and MRI with microsecond dead time. *J. Magn. Reson.* **263**, 147–155 (2016).

10. M. S. Conradi, FET Q switch for pulsed NMR. *Rev. Sci. Instrum.* **48**, 359–361 (1977).

11. A. S. Peshkovsky, J. Forguez, L. Cerioni, D. J. Pusiol, RF probe recovery time reduction with a novel active ringing suppression circuit. *J. Magn. Reson.* **177**, 67–73 (2005).

12. H. T. Stokes, Tuned limiter for receiver amplifier in a fast-recovery pulsed NMR spectrometer. *Rev. Sci. Instrum.* **49**, 1011–1012 (1978).

13. A. C. Özen, E. Atalar, J. G. Korvink, M. Bock, In vivo MRI with concurrent excitation and acquisition using automated active analog cancellation. *Sci. Rep.* **8**, 10631 (2018).

14. D. Idiyatullin, S. Suddarth, C. A. Corum, G. Adriany, M. Garwood, Continuous SWIFT. *J. Magn. Reson.* **220**, 26–31 (2012).

15. D. L. Olson, T. L. Peck, A. G. Webb, R. L. Magin, J. V. Sweedler, High-resolution microcoil ^{1}H-NMR for mass-limited, nanoliter-volume samples. *Science* **270**, 1967–1970 (1995).

16. P. A. S. Cruickshank, D. R. Bolton, D. A. Robertson, R. I. Hunter, R. J. Wylde, G. M. Smith, A kilowatt pulsed 94 GHz electron paramagnetic resonance spectrometer with high concentration sensitivity, high instantaneous bandwidth, and low dead time. *Rev. Sci. Instrum.* **80**, 103102 (2009).

17. R. V. Pound, W. D. Knight, A radiofrequency spectrograph and simple magnetic-field meter. *Rev. Sci. Instrum.* **21**, 219–225 (1950).

18. P. Deschamps, J. Vaissiére, N. S. Sullivan, Integrated circuit Robinson oscillator for NMR detection. *Rev. Sci. Instrum.* **48**, 664–668 (1977).

19. T. Yalcin, G. Boero, Single-chip detector for electron spin resonance spectroscopy. *Rev. Sci. Instrum.* **79**, 094105 (2008).

20. M. Kern, T. Klotz, M. Spiess, P. Mavridis, B. Blümich, J. Anders, Dead time-free detection of NMR signals using voltage-controlled oscillators. *Appl. Magn. Reson.* **54**, 1649–1662 (2023).

21. M. Kern, A. Chu, J. Anders, Current trends in VCO-based EPR. *Appl. Magn. Reson.* **55**, 1065–1089 (2024).

22. A. Chu, B. Schlecker, K. Lips, M. Ortmanns, J. Anders, "An 8-channel 13GHz ESR-on-a-chip injection-locked vco-array achieving 200μM-concentration sensitivity," in *2018 IEEE*

International Solid-State Circuits Conference - (ISSCC) (IEEE, 2018), pp. 354–356; https://ieeexplore.ieee.org/document/8310330.

23. B. Schlecker, A. Hoffmann, A. Chu, M. Ortmanns, K. Lips, J. Anders, Towards low-cost, high-sensitivity point-of-care diagnostics using VCO-based ESR-on-a-chip detectors. *IEEE Sensors J.* **19**, 8995–9003 (2019).

24. M. A. Hassan, T. Elrifai, A. Sakr, M. Kern, K. Lips, J. Anders, "A 14-channel 7 GHz VCO-based EPR-on-a-chip sensor with rapid scan capabilities," in *2021 IEEE Sensors* (IEEE, 2021), pp. 1–4; https://ieeexplore.ieee.org/document/8310330.

25. S. Künstner, A. Chu, K.-P. Dinse, A. Schnegg, J. E. McPeak, B. Naydenov, J. Anders, K. Lips, Rapid-scan electron paramagnetic resonance using an EPR-on-a-chip sensor. *Magn. Reson.* **2**, 673–687 (2021).

26. M. A. Hassan, M. Kern, A. Chu, G. Kalra, E. Shabratova, A. Tsarapkin, N. MacKinnon, K. Lips, C. Teutloff, R. Bittl, J. G. Korvink, J. Anders, Towards single-cell pulsed EPR using VCO-based EPR-on-a-chip detectors. *Frequenz* **76**, 699–717 (2022).

27. A. Chu, M. Kern, K. Khan, K. LiPS, J. Anders, "A 263GHz 32-channel EPR-on-a-chip injection-locked VCO-array," in *2023 IEEE International Solid-State Circuits Conference (ISSCC)* (IEEE, 2023), pp. 20–22; https://ieeexplore.ieee.org/document/10067623.

28. AIST, Spectral database for organic compounds, SDBS; https://sdbs.db.aist.go.jp [accessed 10 February 2026].

29. D. I. Hoult, R. E. Richards, The signal-to-noise ratio of the nuclear magnetic resonance experiment. *J. Magn. Reson.* **24**, 71–85 (1976).

30. A. Chu, B. Schlecker, M. Kern, J. L. Goodsell, A. Angerhofer, K. Lips, J. Anders, On the modeling of amplitude-sensitive electron spin resonance (ESR) detection using voltage-controlled oscillator (VCO)-based ESR-on-a-chip detectors. *Magn. Reson.* **2**, 699–713 (2021).

31. J. Anders, M. Ortmanns, G. Boero, “Frequency noise in current-starved CMOS LC tank oscillators,” in *NDES 2012; Nonlinear Dynamics of Electronic Systems* (IEEE, 2012), pp. 1–4; https://ieeexplore.ieee.org/document/6293767.

32. J. Anders, M. Ortmanns, G. Boero, Noise in frequency-sensitive ESR detectors. *IFAC Proc. Vol.* **45**, 451–456 (2012).

33. J. Anders, “Fully-integrated CMOS probes for magnetic resonance applications,” thesis, EPFL (2011).

34. C. C. Enz, E. A. Vittoz, *Charge-Based MOS Transistor Modeling: The EKV Model for Low-power and RF IC Design* (Wiley, ed. 1, 2006); https://onlinelibrary.wiley.com/doi/book/10.1002/0470855460.

35. J. Anders, “Nonlinear modeling of continuous-wave spin detection using oscillator-based ESR-on-a-chip sensors,” in *Recent Advances in Nonlinear Dynamics and Synchronization: With Selected Applications in Electrical Engineering, Neurocomputing, and Transportation*, K. Kyamakya, W. Mathis, R. Stoop, J. C. Chedjou, Z. Li, Eds. (Springer International Publishing, 2018), pp. 57–87; https://doi.org/10.1007/978-3-319-58996-1_4.

36. S. Bader, M. Ortmanns, J. Anders, “Nonlinear energy-efficient noise-aware design of CMOS LC tank oscillators,” in *2018 IEEE International Symposium on Circuits and Systems (ISCAS)* (IEEE, 2018), pp. 1–5; https://ieeexplore.ieee.org/document/8351618.

37. G. Gualco, J. Anders, A. Sienkiewicz, S. Alberti, L. Forro, G. Boero, Cryogenic single-chip electron spin resonance detector. *J. Magn. Reson.* **247**, 96–103 (2014).
